# The law of flowing water grouting travel and sedimentary plugging under the influence of many factors

**DOI:** 10.1038/s41598-025-90392-7

**Published:** 2025-04-01

**Authors:** Fuyu Wang, Jiafan Zhang, Xiangrui Qin, Huimei Zhang

**Affiliations:** 1 College of Architecture and Civil Engineering, Xi’an University of Science and Technology, Xi’an, 710054 China; 2 Department of Mechanics, Xi’an University of Science and Technology, Xi’an, 710054 China

**Keywords:** 3D printing, Multiple factors, Flowing water grouting, Adversarial modes, Deposition mechanism, Test study, Environmental sciences, Energy science and technology, Engineering

## Abstract

Engineers face significant challenges in determining the grouting parameters and evaluation criteria for environments with flowing water, which has become an urgent matter of concern. Due to the intricate nature of grouting flow and diffusion in practical grouting engineering, numerous fluid-related issues cannot be effectively resolved solely through theoretical analysis or the direct application of fundamental equations. Therefore, it is necessary to employ experimental methods to visually assess and explain these phenomena. In this study, a unique dynamic water grouting test equipment based on 3D printing was designed to evaluate the dynamic grouting travel in cracked rock with flowing water. The influences of fractal dimension, water flow rate, grouting flow rate, and water–cement ratio on the grouting diffusion properties of fractured rock with flowing water were extensively explored. The test demonstrates that the migration and diffusion of grout in the finite boundary fracture can be split into two stages, namely, the circumferent diffusion stage without lateral boundary and the boundary diffusion stage. According to each working circumstance, the diffusion patterns in the process of grouting water plugging are categorized into three types: cross-section, comet, and elongated streamline. A stagnant flow zone was developed in the valley region of the fracture, in which additional particles were deposited. High shear stress was distributed toward the apex area, where few particles were deposited. The experimental results corroborated these observations. The study of the range of grouting diffusion and transport patterns in 3D rough fissures can provide useful insights and guidance for the selection of grouting parameters in grouting engineering practice.

## Introduction

**Fig. 1:**
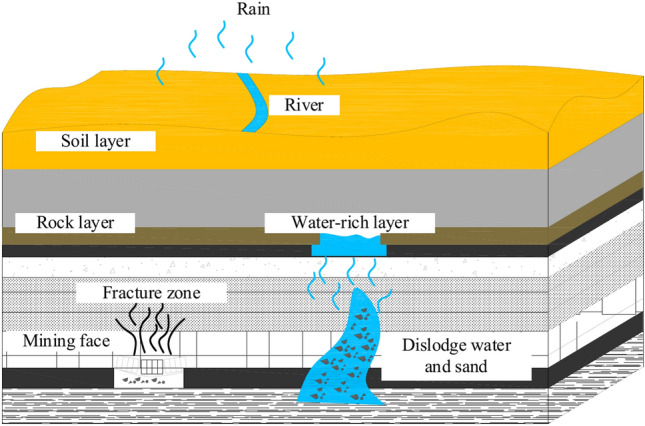
Diagram of geological structure of coal mining^5,6^.

There are thick layers of Jurassic and Cretaceous coal-bearing rock in the mining areas of western China. These layers are weakly adhered to each other, making it very easy for water to break down the rocks. This can also cause water and sand to break through the coal mining face when groundwater and mining pressure work together, as seen in Fig. 1. The roughness of the fracture surface, fracture aperture, fracture network dispersion, and the interaction between grout and groundwater are particularly complex during the grouting and sealing of fractured rock masses in a dynamic water environment, and the flow of non-Newtonian fluids in rock fractures is ubiquitous in engineering practice^1^. As a result, the variable rule of grout pressure, grout diffusion range, distribution method, and sealing effect cannot be successfully predicted. In addition, due to the natural complexity of the fracture surface, current research on fracture dynamic water grouting mostly simplifies it into a two-dimensional smooth plane, ignoring the influence of fracture surface roughness on grout diffusion, resulting in certain deviations between the experimental conclusions and the actual engineering. Conducting field geological and hydrogeological surveys has limits and may yield unclear information for a variety of reasons^2–4^. Due to the complexity of grouting flow and diffusion in practical engineering situations, many fluid problems cannot be handled simply by theoretical analysis or direct application of basic equations,hence, it is important to rely on experimental means for intuitive analysis and discussion.

So far, we have been concerned with the flow between smooth, parallel plates. The roughness of real fracture surfaces introduces tortuosity in the fluid flow and, thus, additional complexity. Fluid flow in a fracture of variable local aperture is a three-dimensional process^7^. Barton^8^ proposed the notion of joint roughness coefficient in the process of researching the structural plane of rock mass, to quantitatively represent the roughness of fracture surface. Later^9^, carried out shear experiments on 136 different rock samples, created 10 classic joint contours according to shear test phenomena, and circumscribed the value range of joint contours (JRC) from 0 to 20 according to the roughness of rock contact. In later investigations, JRC deserves to be extensively recognized by experts all over the world to describe rock mass joints. However, because of the subjectivity of this method, the true characteristics of the rough joints cannot be adequately represented. Subsequently, domestic and foreign experts have conducted quantitative research based on joint shape. Some experts and academics^10–12^ produced the fitting formula of roughness JRC value with the first-order reciprocal root mean square (RMS) and structural function of joint surface by statistical analysis of a large number of rock test data. Hong et al.^13^ evaluated the aliasing effect and roughness characteristics of artificial and natural joint profiles and proposed that at least two roughness factors, such as the amplitude and inclination of the joint, are needed to better reflect the joint roughness. Since the 1980s, with the rapid growth of fractal geometry theory, it has been gradually recognized to utilize fractal geometry theory to describe complicated irregular patterns that are difficult to study by classical geometry^14^. Xie and Zhou^15^ modified the Koch curve to develop a fractal model that can quantify joint surface roughness and discovered the link between the JRC coefficient and fractal dimension D by utilizing regression analysis. Brown^16^ employed two parameters, fractal dimension, and cross length, to define rough surfaces, compared and calculated the contours of fractured rough surfaces using fractal technique and a spectral approach, and studied the influence of roughness on seepage. Chen et al.^17,18^ integrated the fractal theory and the fluctuation value of the joint surface to offer a connection to explain the joint roughness. Therefore, the fractal theory may be utilized to properly characterize the rough cracks in theory and, at the same time, reduce the deviation caused by the subjectivity of the JRC value and the precision of monitoring equipment. The microscopic properties of rough fractures are realized, and the influence of rough fractures on seepage characteristics is reflected.

In the subsequent research, people began to utilize various methodologies to assess the migration mode and dissemination pattern of the grout. Funehaga and Thornb^19^ employed transparent materials to mimic the fracture of rock mass in an anhydrous environment, determined the grout diffusion radius, and compared the measured permeability length with the analytical solution of Bingham grout. Pan et al.^20^ evaluated the effect of coal fragmental size on the law of silica sol grouting and its mechanism. Based on the 2D discrete grouting propagation simulation technique, Mohammed et al.^21^ explored time-dependent hardening of the grout viscosity, initial yield stress of the grout fluid, and the rheology parameters of the in-situ pore fluid. Guo et al.^22^ constructed a theoretical model for grouting into a fracture with flowing water considering the time-varying viscosity of the slurry. Eriksson et al.^23^ employed numerical simulations of grout dispersion and sealing effect for predictions of the grouting result. A novel numerical approach^24^ for grouting in flowing water based on the Euler–Euler framework: two-fluid tracking (TFT) method to handle the problem of unequal distribution of slurry viscosity in time and space during grouting. Wang et al.^25^ established the dynamic water plugging criterion of the glass fiber cement slurry, and the flow pattern test is carried out for the cement slurry and consolidation body with varied glass fiber proportions. For the sealing problem of water rushing in karst pipes, the sealing mechanism and method are examined by numerical simulation, laboratory experimentation, and engineering application^26^. Wang et al.^27,28^ carried out a series of studies on the diffusion mechanism of parallel plate fracture grouting, and carried out studies with and without water. On the basis of theoretical research, a numerical simulation method of rough fracture grout propagation refinement based on Bingham–Papanastasiou rheological model is established^29^. Wang et al.^27,28,30^ studied the mechanical behavior of splitting grouting with solid by using acoustic emission technology and theoretical analysis. The above studies only investigate the fluctuation of roughness of slab fractures or fractures in a two-dimensional plane, but do not consider the effect of three-dimensional rough fractures present in real rock mass on the grouting results.

In summary, many scholars around the world have carried out in-depth research on the theory, materials, numerical simulation, and experimental methods for dynamic water grouting, which has greatly promoted the development of the theory and technology of dynamic water grouting, but there is still no report on the study of the deposition and transport of the slurry under the interaction of multiple factors in the flowing water environment. With the evolution of 3D printing technology, manually replicating actual rough fractures is no longer an intractable issue. This work is based on the fractional Brownian motion to build a three-dimensional rough fracture surface, the use of Rhinoceros software to achieve the generation of three-dimensional data of the rough fracture surface and the establishment of the test model, and to obtain the physical model of the rough fissure surface through 3D printing technology, which gives a model basis for the 3D rough fracture grouting with flowing water test. Afterward, an indoor test system was used to investigate the diffusion law of grout in the fractures of rock bodies with different roughness under the flowing water environment and to verify the feasibility of grouting applied to the seepage control and reinforcement of coal seams under the flowing water environment. The results of the study are of substantial practical importance for the improvement of the quality of the coal bed seepage control and reinforcement project, the advancement of the grouting technology, and the further popularization and deployment of the grouting process. It can achieve the purpose of retaining groundwater and reducing environmental damage.

## Test

### Background of the Balasu mine

Yuheng North District is situated in the central part of the Jurassic coal field in northern Shaanxi Province, China. The district encompasses planned coal mine fields spanning an area of 150–300 km^2^. These fields contain numerous coal seams that can be extracted, offering significant potential for development and substantial economic benefits in the coming decades. Additionally, they hold a crucial strategic position within the coal production base of northern Shaanxi Province. Recently, the coal seams have experienced varying degrees of water damage, including uncommon occurrences of high-pressure water damage in both the Chain and the world. The Balasu well field No. 2 exhibits various patterns of coal seam fissure growth, with closely interconnected hydraulic communication linkages, and the presence of a localized region with high pressure and abundant water. The Balasu well field is located in a Cretaceous clastic pore-fracture aquifer system, so it is essential to discuss the effectiveness of slurry water plugging in rough fractures. Regarding mine water control, it is crucial to prioritize safe production in coal seams with abundant water. This should be followed by efficient coal mining and rational utilization of water resources in the coal seam. Grouting water-plugging holds significant theoretical importance and practical value.

### Test scheme

**Fig. 2:**
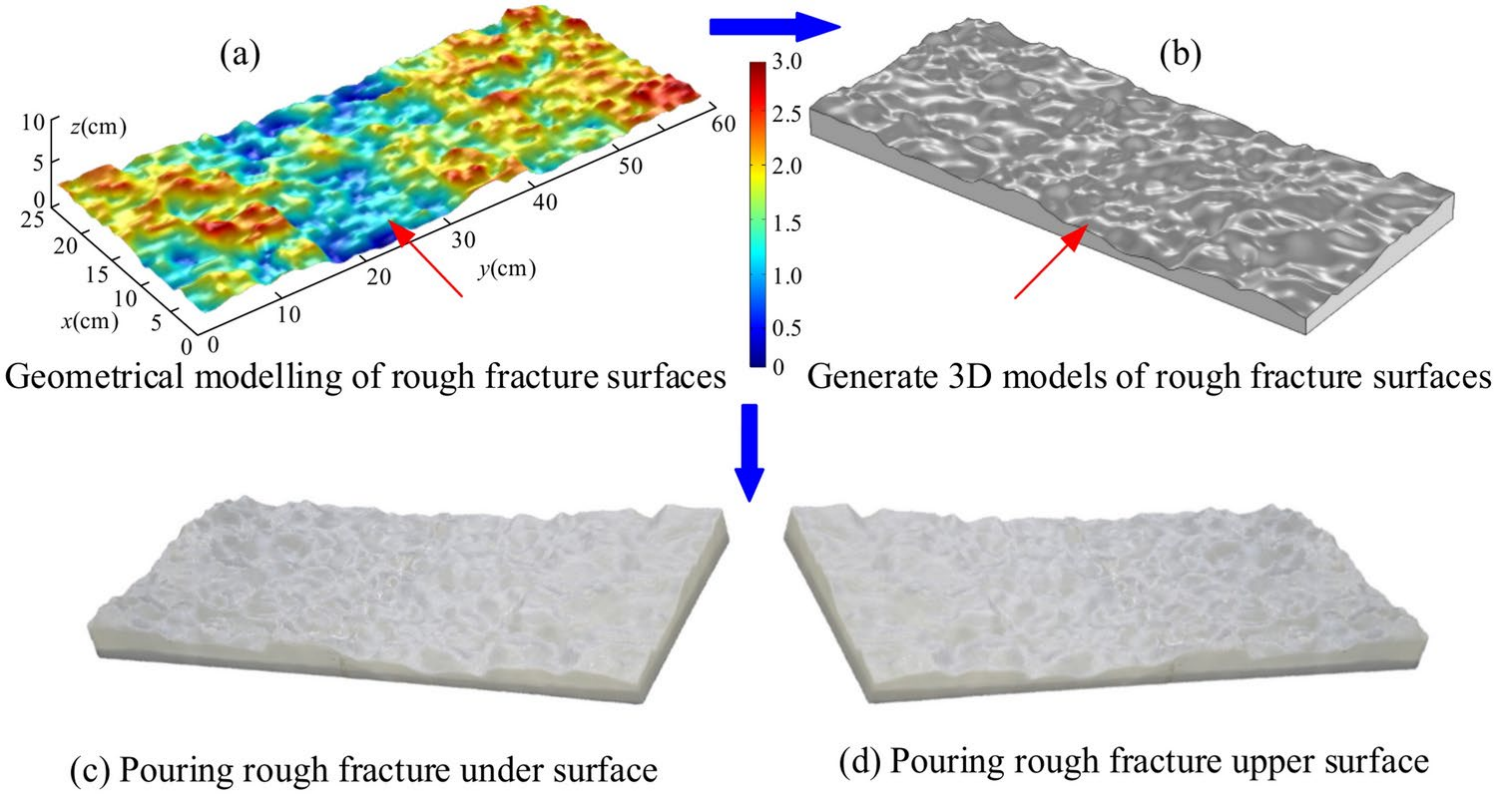
3D printing rough fracture model preparation process.

The dimensions of the rough fissure model were established as 600 mm × 250 mm, taking into account the statistical characteristics of over 200 water-conducting fissures within the roadway enclosure of the Balasu coal mine^31^. The rough fracture simulator is an integral aspect of the rock fracture dynamic water grouting test setup. For this aim, a rough fracture surface^32^ was initially constructed based on the midpoint displacement approach, see Fig. 2a, which consists of a series of data points. The data points of the rough surface were then imported into Rhinoceros software to generate a 3D conceptual model of the rough fracture, see Fig. 2b. Finally, a HORI-z600 3D printer was used to build the preliminary fracture conceptual model. The 3D printer employed has a printing accuracy of 0.01 mm, which ensures the accuracy of the 3D-printed fracture model. The material employed in the 3D printer is polylactic acid (PLA) with great transparency, which fits the parameters for viewing the fracture model. The 3D printed model of the finished fracture was cast with epoxy resin to ensure airtightness, and the top and lower sections are illustrated in Fig. 2c,d. Rough fracture models with fractal dimensions of 2.0, 2.3, 2.5, and 2.7 are constructed similarly to the aforesaid procedure.

**Fig. 3:**
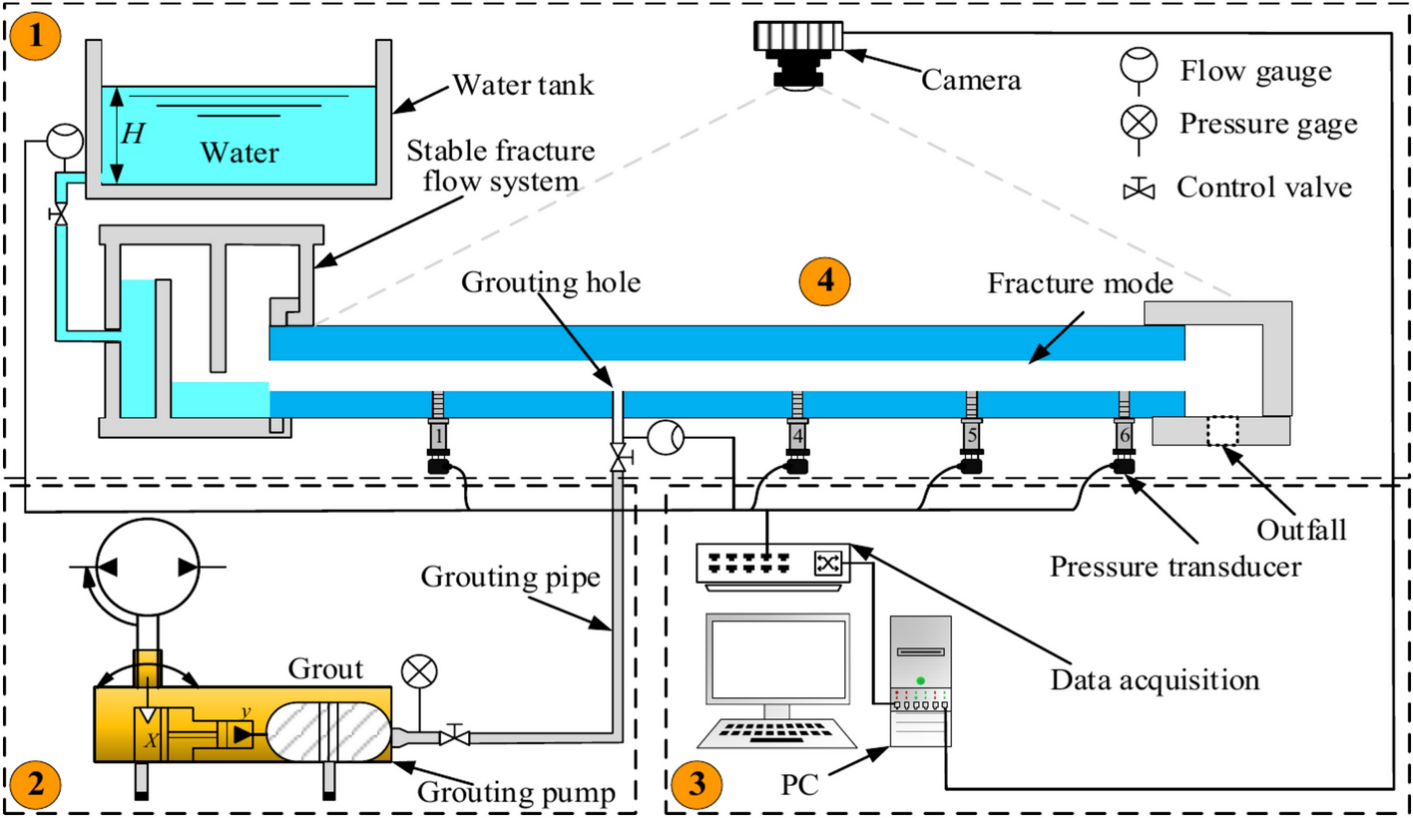
Schematic diagram of the dynamic water grouting system for rough fracture.

According to the self-developed and built dynamic water grouting test system, indoor grouting model tests are carried out to research the grout diffusion law and sealing mechanism during dynamic water grouting. The test system consists of four parts: a fracture simulation test bench (600 × 250 mm), a water supply system, a grouting system, and an image monitoring and data collection system, as illustrated in Fig. 3.

**Fig. 4:**
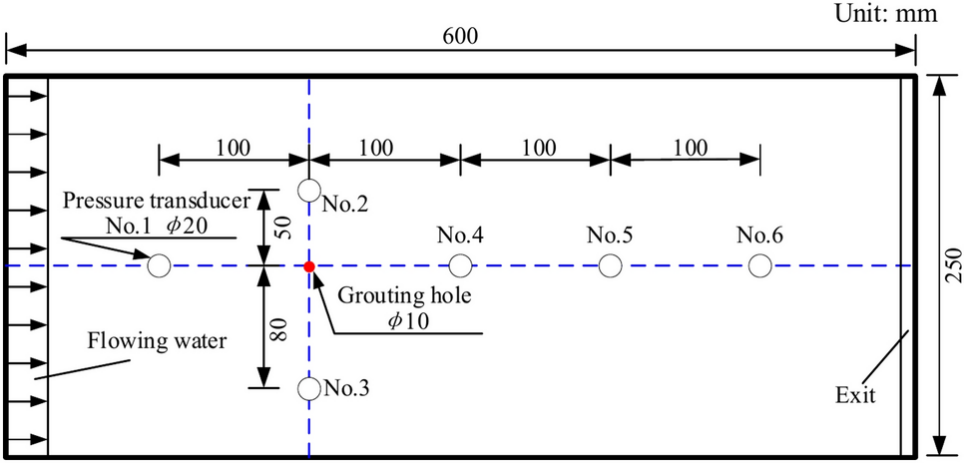
Pressure transducer layout diagram.

This test chose a grout-specific surface area of 600–800 m^2^/kg, fineness to meet the 80 μm square hole sieve, and sieve residue of less than 5%, so the slurry can enter the small cracks. The fracture aperture was set at 3 mm throughout the test, and the test temperature was 20 °C. The specific test steps are as follows:

1. The cement-based grout and water are weighed first according to the water-cement ratio of the test design, and then the water is slowly poured into the mixing drum and, at the same time, swirled with a mixer to make it entirely dissolved;
2. Before starting the test, check whether the test bench system is intact. After establishing that there is no fault, we may reach the design criteria through leveling;
3. Installation of the picture shooting system and connection of the data lines to the acquisition system, the pressure transmitter setup is depicted in Fig. 4;
4. In advance, the rough fracture model is filled with water through the dynamic water simulation system and the height of the water tank is adjusted to make the flow of the dynamic water flow meter indicate the design value;
5. The pre-prepared slurry is churned again and dumped into the slurry bucket. After shutting the valve of the input pipe, remove the slurry pipe joint at the grouting hole, open the valve of the slurry pipe, adjust the height of the slurry bucket so that the slurry flow meter shows the design value, close the valve of the slurry pipe, and finally connect the slurry pipe joint;
6. After pressing the button of the image shooting and data acquisition equipment, open the valve of the inflow pipe and the grouting pipe joint to carry out the grouting test;
7. Close all valves after the test, and remove the cleaning test stand and grouting pipe immediately.

In this section, the diffusion and sealing characteristics of cement-based grout under the influence of several parameters are explored experimentally. The fracture roughness is the smoothness of the fracture, which affects the spreading range and flowing pattern of the grout. Grouting flow rate affects grout flow pattern and grouting pressure. The variation of dynamic water flow also impacts the diffusion and deposition morphology of the grout. The water-to-cement ratio determines the viscosity of the grout. In a real grouting project, in order to ensure the fluidity of the slurry, the water-cement ratio is frequently more than 0.6. Therefore, in this paper, we consider four main factors affecting the grouting effect during dynamic water grouting: fractal dimension (2.0, 2.3, 2.5, 2.7), grouting flow rate (0.06L/s, 0.08L/s, 0.10L/s, 0.12L/s), dynamic water flow rate (0.04L/s, 0.06L/s, 0.08L/s, 0.10L/s), and water-cement ratio (0.6, 0.8, 1.0, 1.2). The test setup is provided in Table 1, with a total of 13 working situations to explore the influence of different elements on the test results, respectively.

**Table 1:** Rough fracture dynamic water grouting test table.

Serial number	Fractal dimension/D	Dynamic water flow rate/Q_w_ (L·s^−1^)	grouting flow rate/Q_J_ (L·s^−1^)	Water–cement ratio W/C
1#	2.0	0.04	0.06	1.0
2#	2.3			
3#	2.5			
4#	2.7			
5#	2.7	0.06	0.06	1.0
6#		0.08		
7#		0.10		
8#	2.7	0.1	0.08	1.0
9#			0.10	
10#			0.12	
11#	2.7	0.06	0.06	0.6
12#				0.8
13#				1.2

### Analysis of test results

**Fig. 5:**
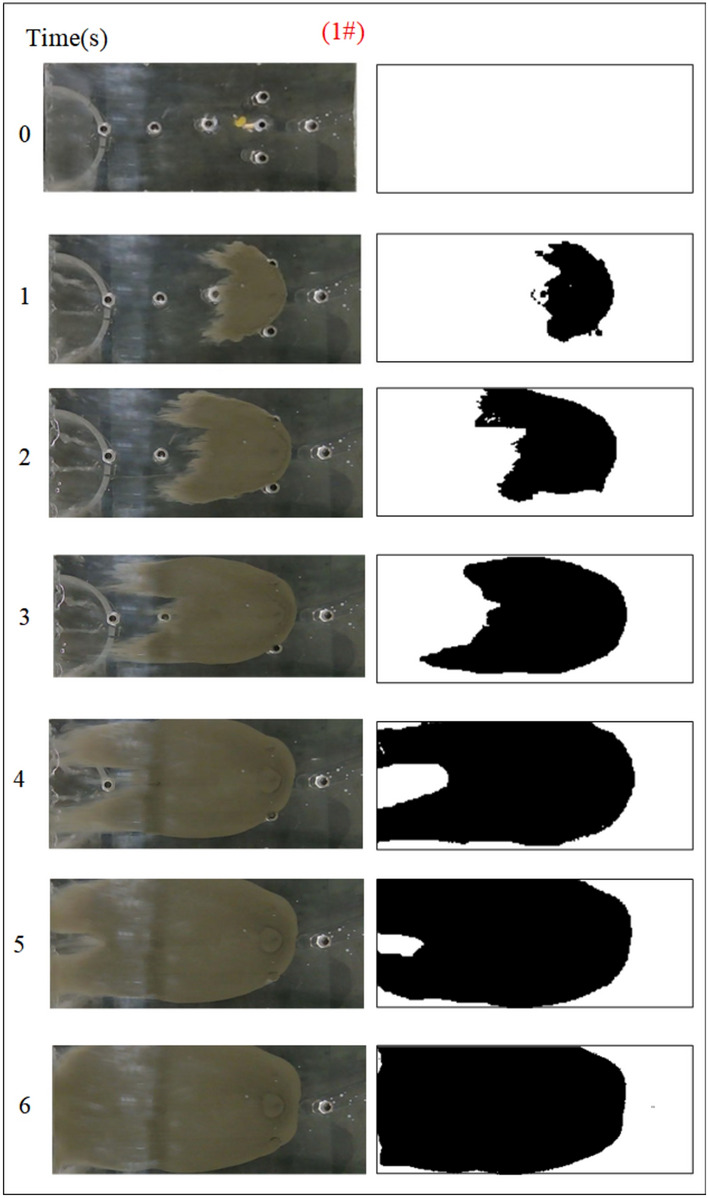
Grout diffusion patterns at D = 2.0

**Fig. 6:**
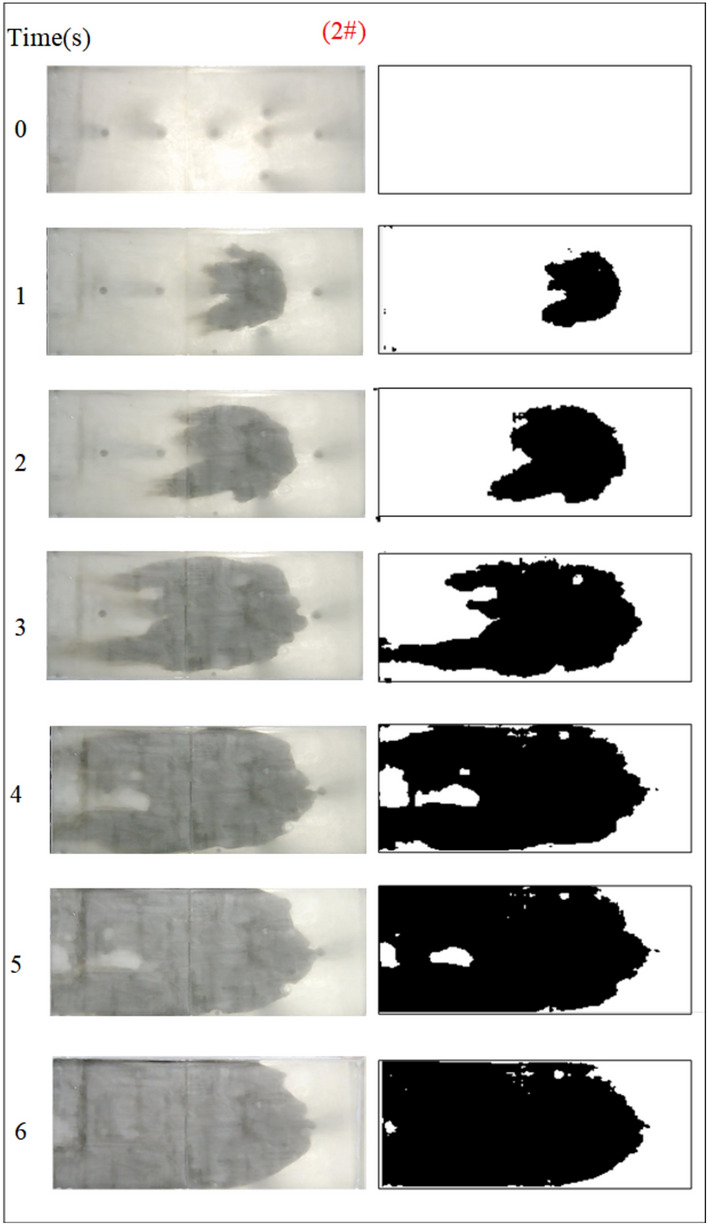
Grout diffusion patterns at D = 2.3

**Fig. 7:**
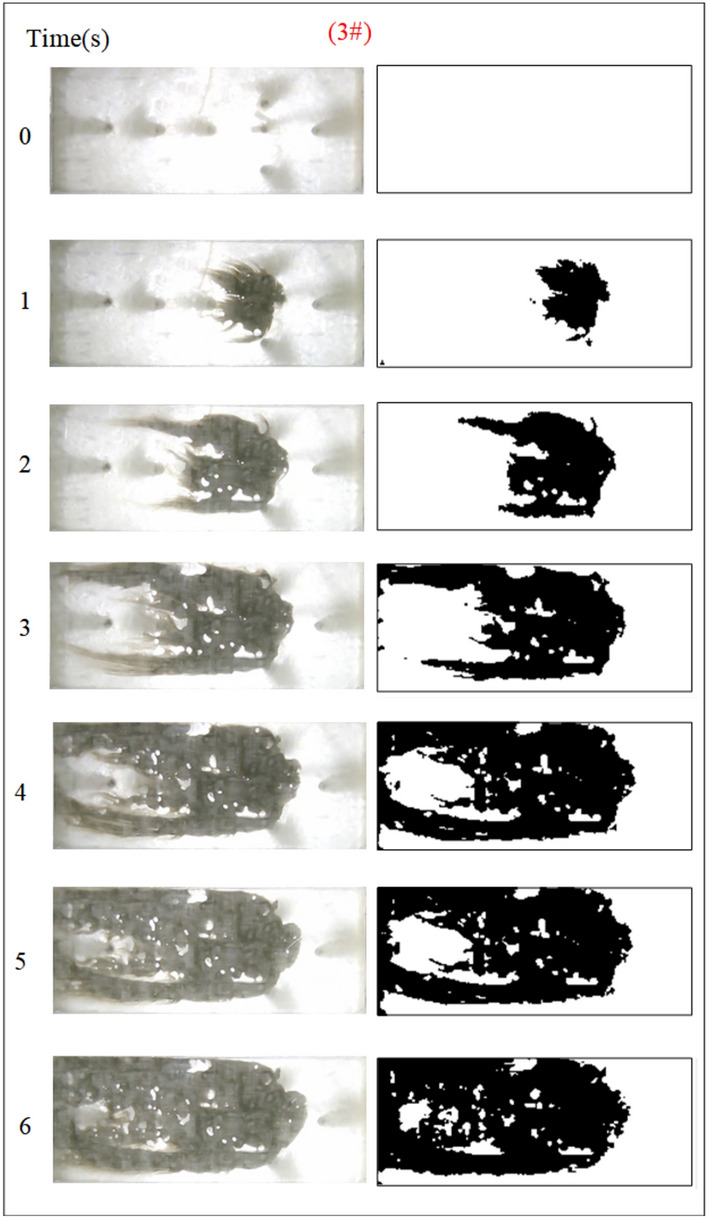
Grout diffusion patterns at D = 2.5

**Fig. 8:**
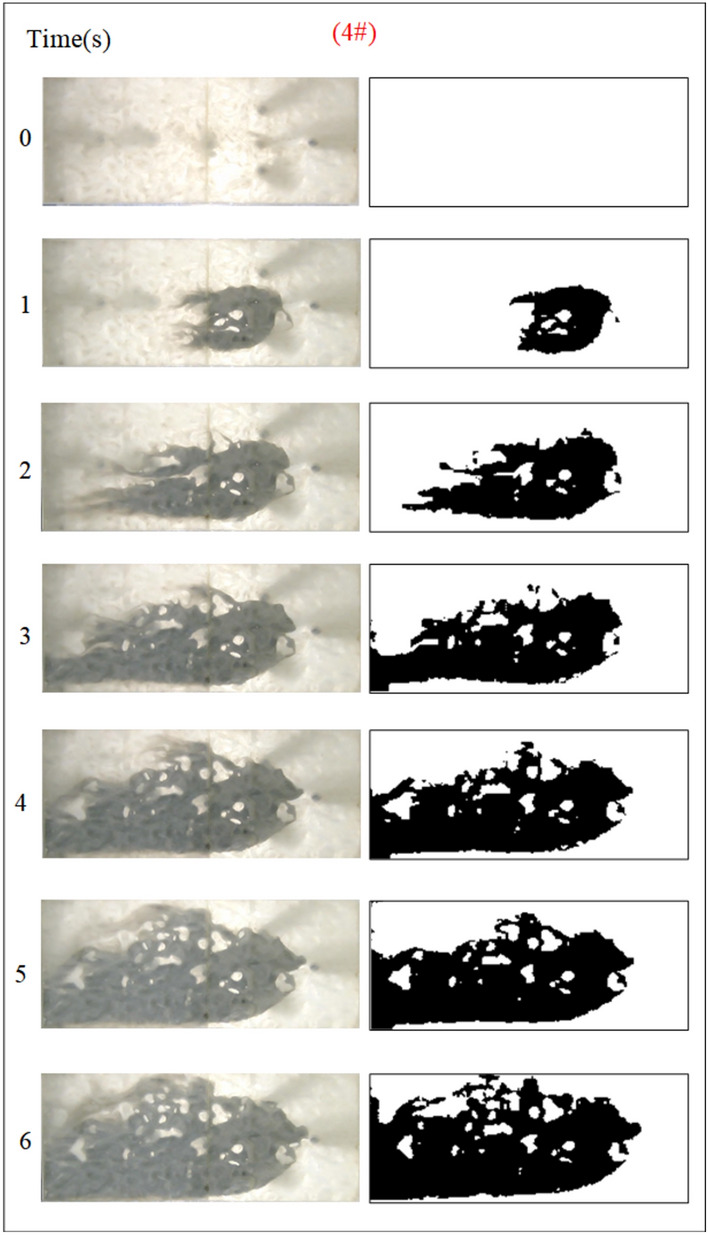
Grout diffusion patterns at D = 2.7

The influence of the fractal dimension of the fracture on the spreading range and flow pattern of the grout is explored while guaranteeing that the rest of the parameters remain unchanged. The test numbers were 1#, 2#, 3#, and 4# accordingly, and the grout diffusion morphology was illustrated in Figs. 5, 6, 7, 8. The grout is injected into the fracture, and the grout spreads radially in a circular shape in all directions at first. However, due to the action of dynamic water, the diffusion speed upstream is smaller than the downstream diffusion speed. The upper reaches of the grouting hole produced an uneven, semi-circular shape under the strong battle between the grout and flowing water. After the grout diffused away from the grouting hole on the left and right sides, it was highly impacted by the impact of flowing water and began to move downstream, and the migration speed was greater than the grout diffusion speed along the center line under the operation of grouting pressure. As a result, the grout on the downstream side of the fissure begins to form a concave upstream arc. As the grout injection rises, the grout spreading upstream is continually transported downstream by the water, and due to the existence of the fracture boundary, the downstream grout continuously accumulates and fills the fracture.

Due to the varied fractal dimensions of the fracture surface, the spreading pattern of the slurry also appeared to shift. Comparison of Figs. 5, 6, 7, 8 reveals that the region of grouting diffusion is growing with time. The grout spreading along the backwater direction is prevented by the flowing water, while the grout spreading along the downstream direction is driven by the flowing water, which results in the grout spreading distance against the water being smaller than the distance spreading along the water. Grout diffusion fronts in smooth fractures are typically circular and more regular, with the diffusion cover becoming more irregular as the roughness rises. This shows that the rough fracture surface impacts the spreading of the grout, and the spreading trail of the grout in the rough fracture is not uniform along all directions. In addition, the contrast with the smooth fracture grouting pattern is the presence of a significant water cavity region in the rough fracture, where no grout is formed. Wang et al.^5,6^ also discovered such water cavity zones in their research of grout-groundwater interactions in rough fractures. The difference is that in our investigation, it was found that the bigger the fractal dimension of the fracture, the larger the size of the water cavity, and with the continuous diffusion of the grout zones, a portion of the water cavity was gradually filled or reduced by the grout.

**Fig. 9:**
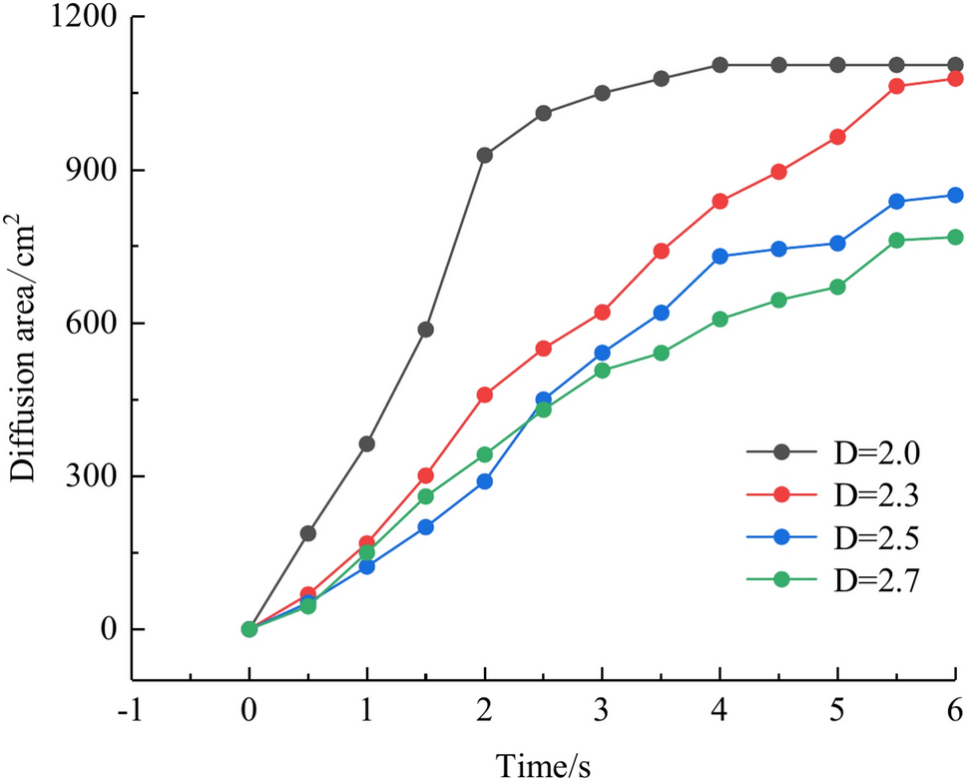
Area of grout diffusion in the fracture with different fractal dimensions.

To quantitatively assess the effect of fracture fractal dimension on the morphology of grout diffusion, as illustrated in Fig. 5, the grout diffusion image was first identified in the grayscale. The grayscale image matrix is then subjected to a binary transformation process to obtain a digital matrix consisting of 0 and 1, which is presented as a binarized image with a sharp contrast between black and white; matrix element 0 is in black to indicate grout settling and element 1 is in white to indicate the water flow channels and water cavities. Finally, the area of the grout diffusion region is calculated by counting the ratio of element 0 in the binarized matrix to all matrix elements, as illustrated in Fig. 9. The grout diffusion area demonstrates a non-linear growth change with the grouting time. In the early stage of grouting, the diffusion area increases rapidly and the diffusion speed is the fastest; with the subsequent growth of grouting time, the diffusion speed slows down progressively until the grout achieves the maximum diffusion area and the grout diffusion range is no longer increased. As shown in Fig. 9, the maximum diffusion area of the grout at fractal dimensions D = 2.0, 2.3, 2.5, and 2.7 is 1105.5 cm2, 1078.5 cm2, 850.5 cm2, and 768 cm2, with declines of 2.44%, 20.86%, and 9.70%, respectively. It may be found by comparison that the higher the fractal dimension of the fracture, the longer the time to achieve the maximum diffusion region. The above phenomenon reveals that the fractal dimension of the fracture has a considerable effect on the grout diffusion (area and diffusion time), and with the growth of the fractal dimension, the effect is more pronounced.

**Fig. 10:**
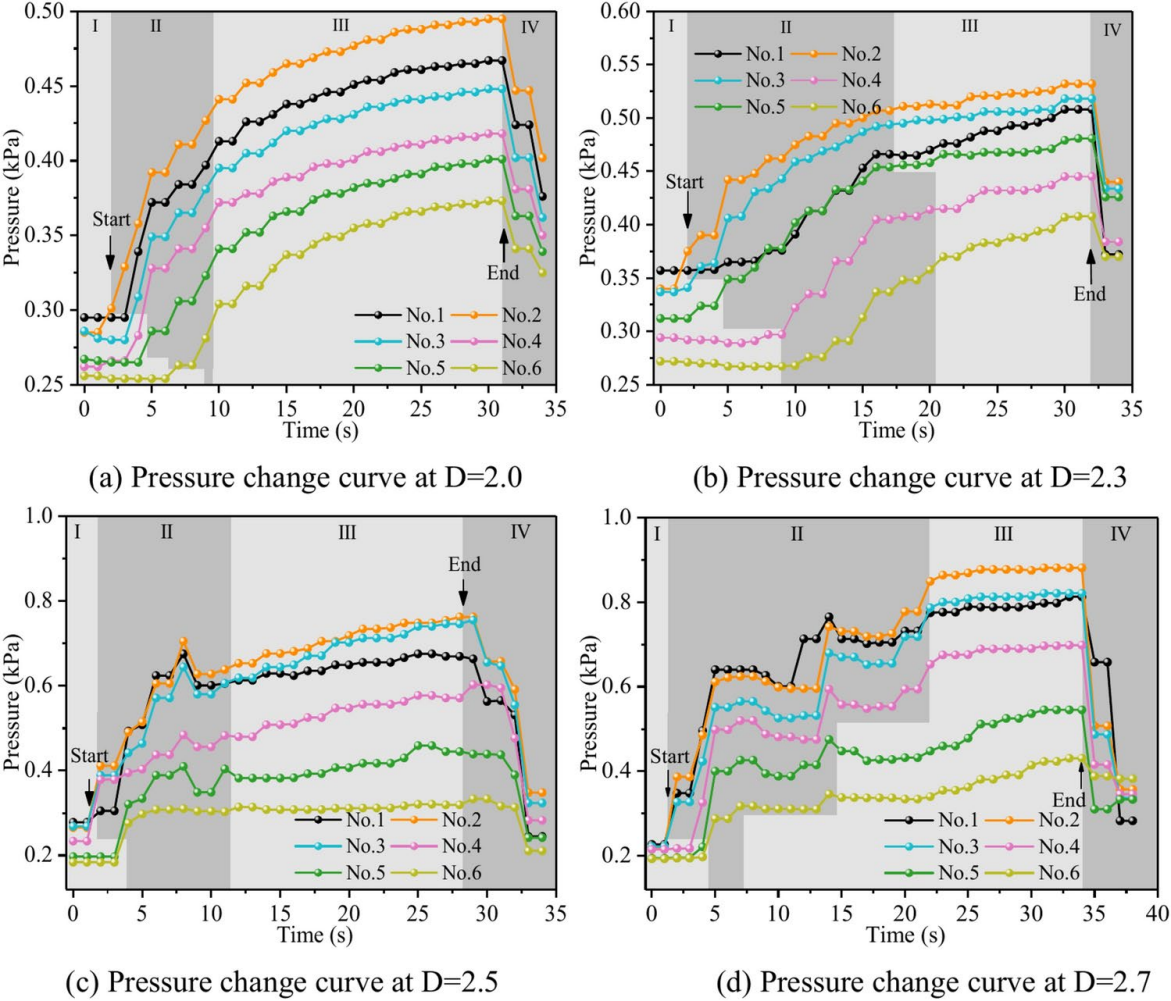
Pressure data at each monitoring point under different fractal dimensions.

**Fig. 11:**
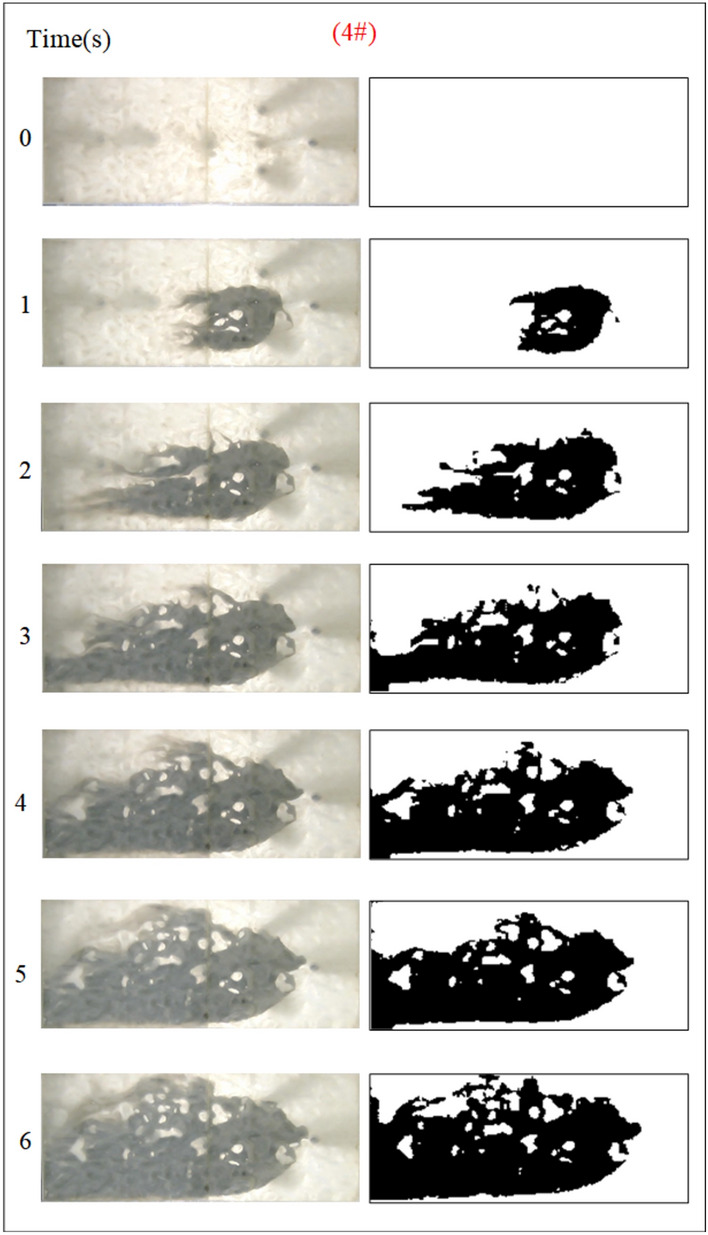
Grout diffusion patterns at Q_w_ = 0.04L/s.

Figure 10 depicts the fluctuation in fluid pressure within the fractures during the grouting operation in Cases 1# to 4#. In this research, the pressure change in the process of dynamic water grouting is separated into four stages: Stage I is the stage of dynamic water injection; Stage II is the stage of slurry injection when the pressure increases rapidly; Stage III is the stage of grout diffusion, and the pressure is gradually stabilized at a constant value; and Stage IV is the stage of stopping grouting when the grout diffuses to the boundary of the fracture model to stop grouting. The change in pressures depends not only on the location of the measuring points but also on the sealing and gelling conditions and the type of liquids at those places (water, grout, or water and grout mixture). In general, fluid pressure increases with time in the region of grout buildup until grouting stops. The peak pressures within the fractures at fractal dimensions D = 2.0, 2.3, 2.5, and 2.7 were 0.495 kPa, 0.532 kPa, 0.762 kPa, and 0.813 kPa, with pressure increases of 7.5%, 43.2%, and 6.7%, respectively. As can be seen from Fig. 11, the smaller the fractal dimension, the smoother the pressure profile within the fracture. The explanation is that, as the fractal dimension increases, the degree of zigzagging of the fracture is greater, and the intensity of the confrontation between grout and water in the fracture is amplified, such that there is a phenomenon of “stepwise” increase in the pressure value. The explanation is that, as the fractal dimension grows, the degree of zigzagging of the fracture is greater, and the intensity of the confrontation between grout and water in the fracture is enhanced, such that there is a phenomenon of a stepwise increase in the pressure value. Taking the fractal dimension of 2.0 as an example for analysis, the peak pressures of Nos. 1 ~ 6 sensors are 0.467 kPa, 0.495 kPa, 0.448 kPa, 0.418 kPa, 0.401 kPa, and 0.373 kPa, respectively, and the above pressure data can illustrate that the farther away from the grouting hole, the less pronounced the decrease in the pressure value of the sensors, and the less grout is deposited.

**Fig. 12:**
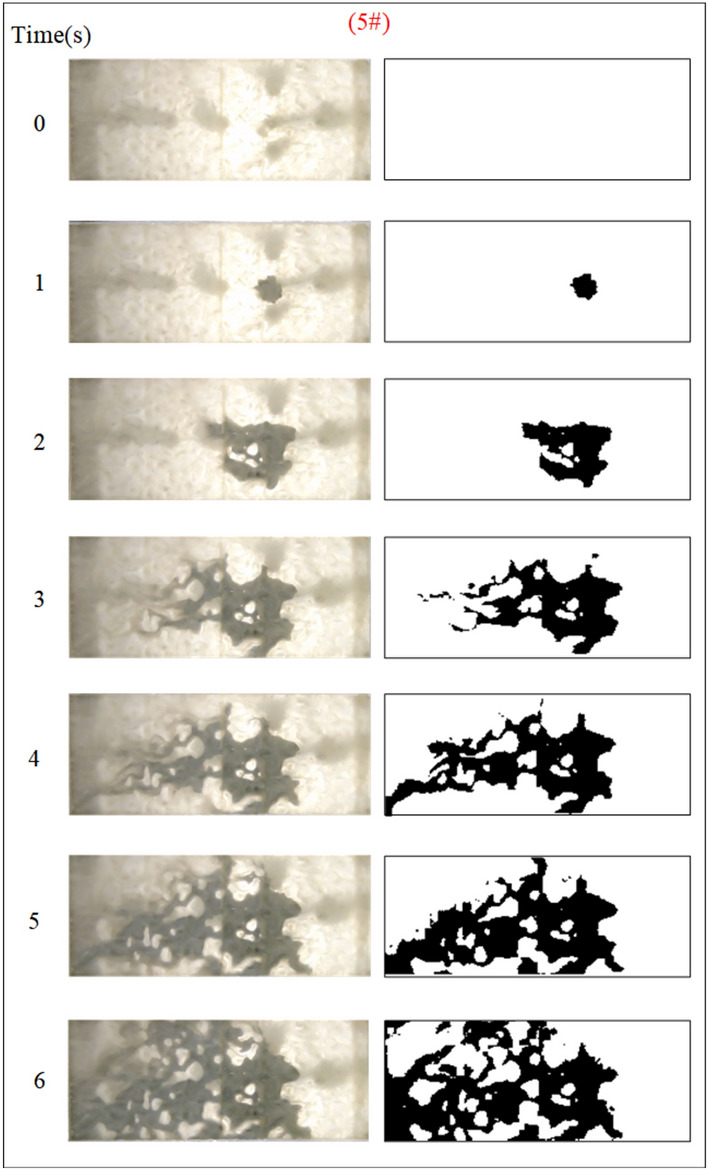
Grout diffusion patterns at Q_w_ = 0.06L/s.

**Fig. 13:**
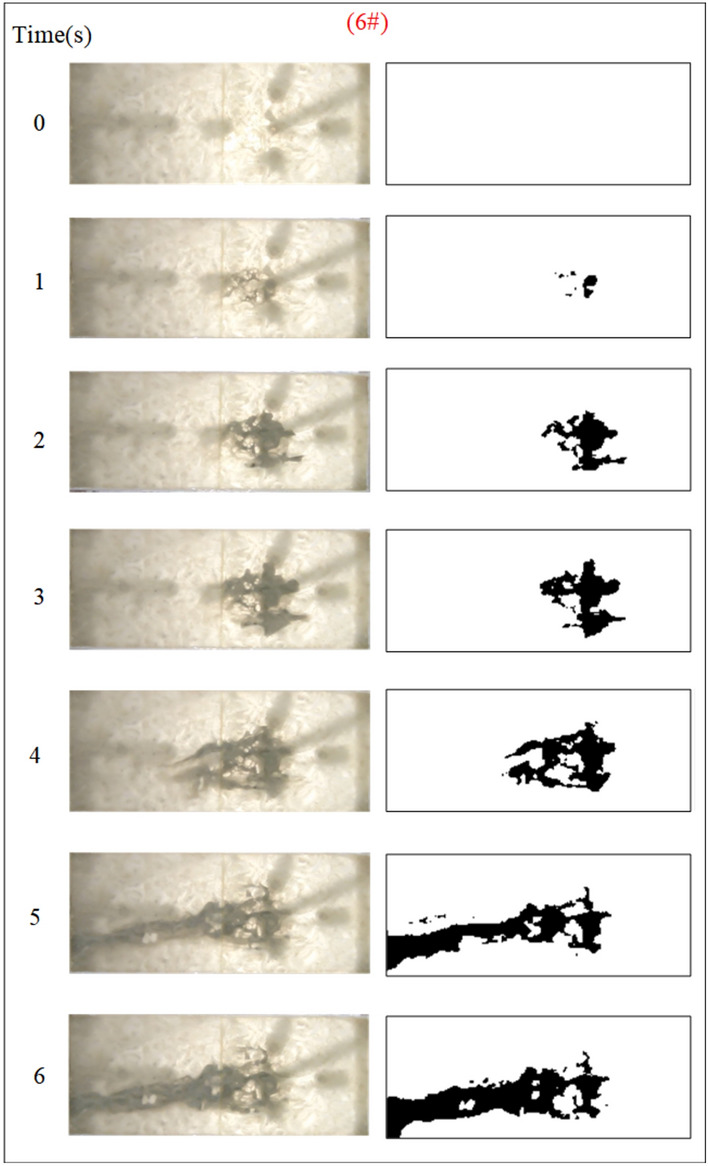
Grout diffusion patterns at Q_w_ = 0.08L/s.

**Fig. 14:**
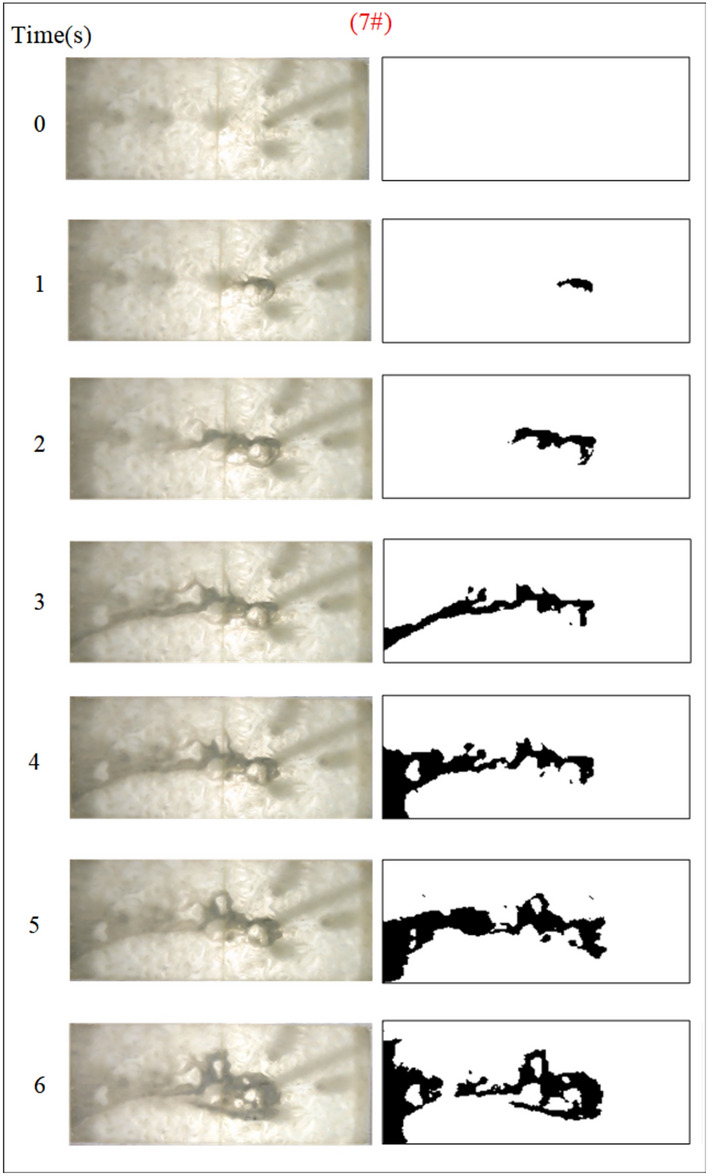
Grout diffusion patterns at Q_w_ = 0.10L/s.

Under the condition of guaranteeing that the rest of the parameters remain unchanged, the influence of the fracture dynamic water flow rate on the spreading range and flow pattern of the grout is explored. The test numbers are 4#, 5#, 6#, and 7#, and the grout dispersion patterns are illustrated in Figs. 11, 12, 13, 14. The grout is injected into the fracture; initially, the grout spreads radially in all directions, but due to the roughness of the fracture, water cavity blisters are generated throughout the grouting process, and the grout has a roughly irregular circular shape. Under the effect of flowing water, the grout diffusion velocity towards the water is smaller than that following the water. Upstream of the grout in the grouting pressure and flowing water under the role of intense confrontation to form an irregular semicircular shape, the left and right sides of the slurry diffusion away from the grouting hole, by the strong impact of the dynamic water impact, began to move downstream, and the speed of transport is greater than near the center line of the slurry diffusion speed under the action of the grouting pressure. With the rising grout injection volume, the grout spreading upstream is continually carried downstream by the flowing water, and due to the existence of the fracture border, the downstream slurry continuously accumulates and fills the fracture.

The grout diffusion pattern under the action of dynamic water is extremely different from the previous operating situation, and various intriguing events occurred. According to the experimental phenomena in Figs. 11, 12, 13, 14, after examining the relationship between the dynamic water flow rate and the grouting flow rate, they can be divided into three types of diffusion patterns. (1) When the dynamic water flow rate is less than the grouting flow rate, at this time, due to the small dynamic water flow rate, the grout in the fracture is in a planar propulsion state, and the grout does not cause a wide range of confrontation with the water. With the rise in time, grout gradually fills the fracture, but there are a high number of water cavities, and the end of the fracture grout-water mixture outflow is extremely tiny. Downstream is filled with grout, and the grout is progressively moved upstream; (2) When the dynamic water flow rate is equal to the grouting flow rate, the two sides of the formation form a clean water channel. Grout is offered as a cross-section type, and the development of the water cavity is tiny; (3) When the dynamic water flow rate is higher than the grouting flow rate, it is difficult for the grout to be kept in the fracture, and a substantial volume of grout is washed away by the dynamic water. Eventually, the grout only survives in the grouting hole and the end of the fracture, and between the two ends of the grout diffusion pattern for the elongated slim-line type, there is no water-plugging effect at all.

**Fig. 15:**
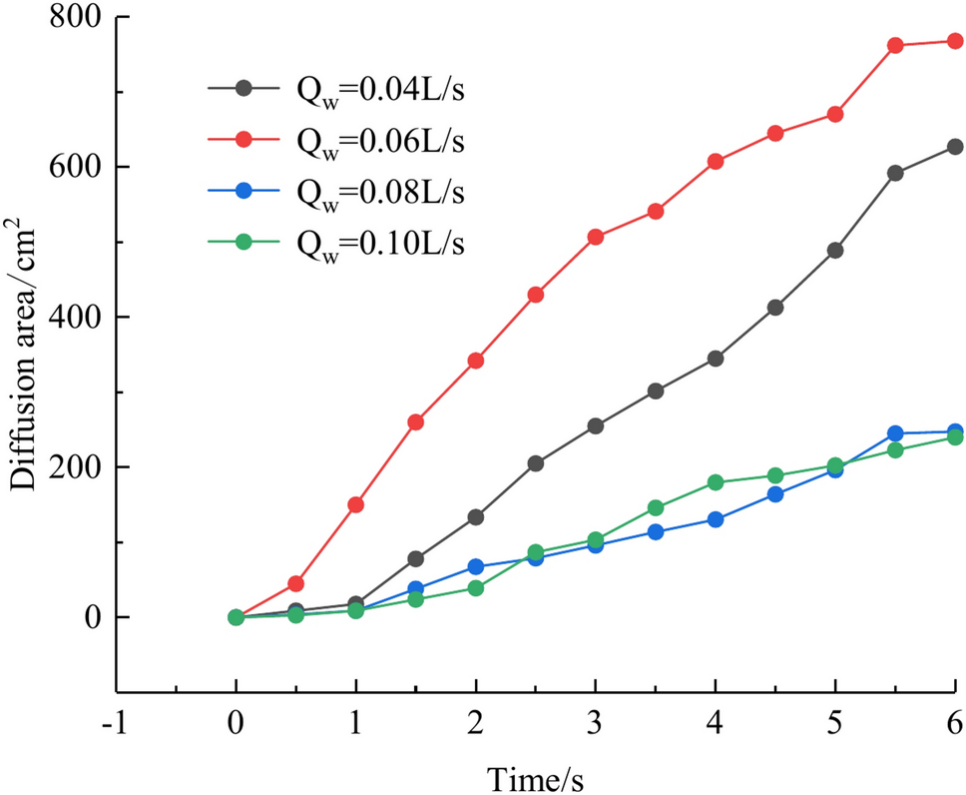
Grouting diffusion area in the fracture under different dynamic water flow rates.

Based on the grayscale identification approach above, the grout diffusion area at different dynamic water flow rates is computed as shown in Fig. 15. The maximal diffusion area of the grout at the dynamic water flow rates Qw = 0.04 L/s, 0.06 L/s, 0.08 L/s, and 0.10 L/s is 768 cm^2^, 627 cm^2^, 247.5 cm^2^, and 240 cm^2^, respectively. Test results demonstrate that if the dynamic water flow rate is greater than the grouting flow rate, it cannot reach the expected diffusion area and cannot be effective for water plugging. If the dynamic water flow rate is less than or equal to the grouting flow rate, the effect of water plugging is visible. It was also revealed that when the dynamic water flow rate was minimal (in the interval of the dynamic water flow rate less than or equal to the grouting flow rate), there were a large number of water cavities, which were unfavorable to the strength increase in the latter stage. The rationale for the analysis is that the cavity formed in the grouting process cannot be excluded in time, and the running water has almost no antagonism with the grout. The occurrence of a large number of grout particles seeping into the water column without sufficient pressure to fill the gaps may also be recognized by comparing Figs. 11 and 12.

**Fig. 16:**
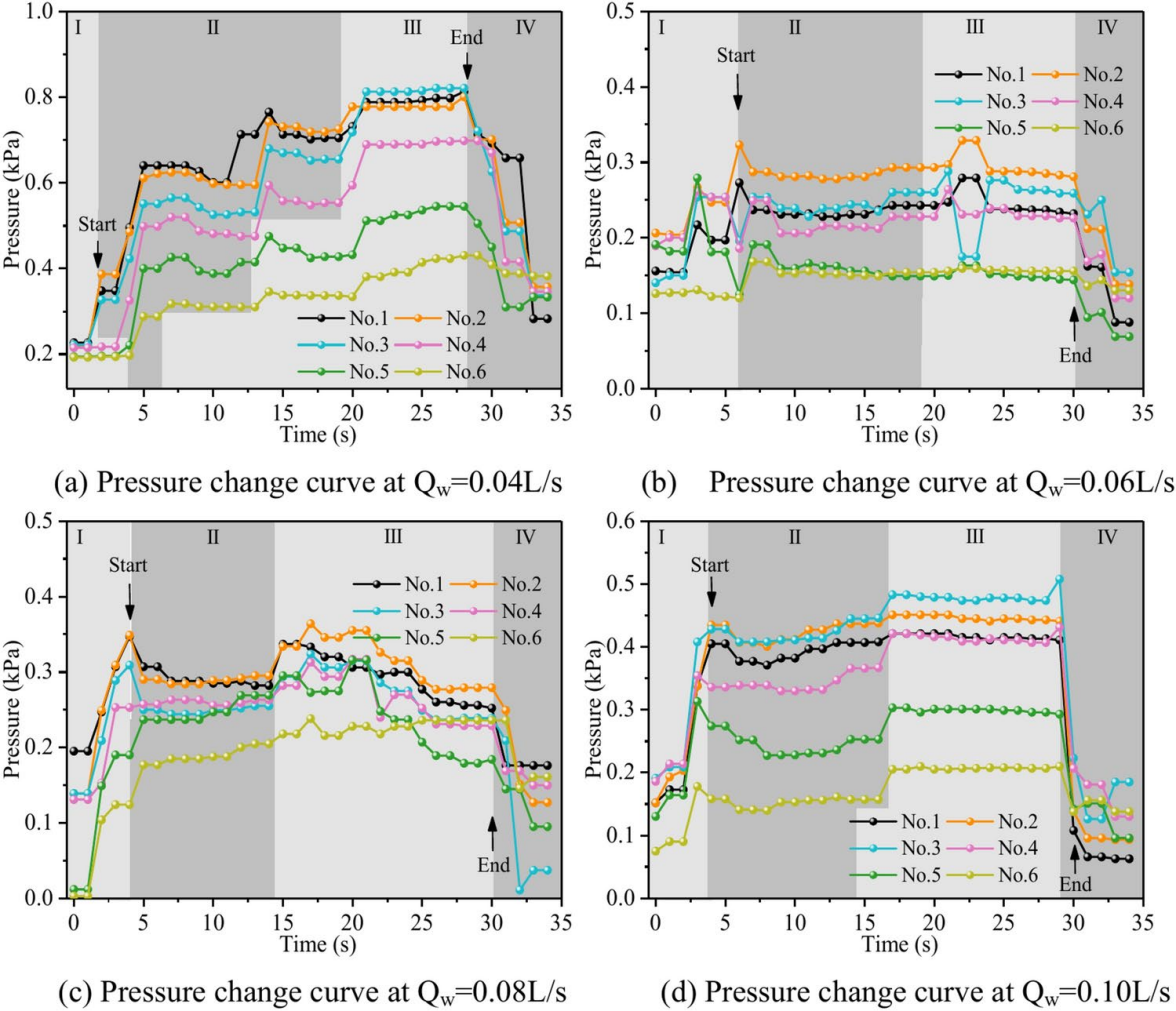
Pressure data at each monitoring point under different dynamic water flow rates.

Figure 16 shows the variation of fluid pressure within the fractures during the grouting process in Cases 4# to 7#. In general, at the location of grout buildup, the fluid pressure increased with time until the grouting stopped. The peak pressures in the fractures at dynamic water flow rates Qw = 0.04 L/s, 0.06 L/s, 0.08 L/s, and 0.10 L/s were 0.821 kPa, 0.329 kPa, 0.355 kPa, and 0.508 kPa, respectively. The explanation for the excessively high fracture pressure in Case 4# is that grouting deposits collect and impede the major flow channel. As can be observed in Fig. 16, with the growth of the dynamic water, the severity of the confrontation between the grout and water within the fracture intensifies, and a fluctuating increase in the pressure values occurs as a result. When the dynamic water flow rate is less than or equal to the grouting flow rate, the upstream grout and water impede each other, while the downstream water is pressed away by the grout, generating a comet-shaped structure. As can be observed in Fig. 16, with the growth of the dynamic water, the severity of the confrontation between the grout and water within the fracture intensifies, and a fluctuating increase in the pressure values occurs as a result. At the upstream interface, the movement of water and grout cancels each other out and reaches a stationary state. The grout first gels at the front face, where the flow velocity is lowest and a retention nucleus (stagnation area) is created. The grout circulates in the vicinity of the stagnation area and then continues to cement. As the area of cementation develops and eventually reaches the margin of the fracture, water flow is halted, as shown in Fig. 11. If the grout does not spread to the border, the water will continue to flow along both sides of the passage, as shown in Fig. 12. When the dynamic water flow rate is larger than the grouting flow rate, the water and grout are in fierce confrontation, and the water flushes the grout so that it cannot be retained in large quantities to form a stagnation area within the fracture and ultimately exists in the state of elongated streamlines, as shown in Figs. 13 and 14. Taking the dynamic water flow rate of 0.04L/s as an example for analysis, the peak pressures of Nos. 1 ~ 6 sensors are 0.813 kPa, 0.801 kPa, 0.821 kPa, 0.695 kPa, 0.545 kPa, 0.431 kPa, respectively. Due to the mutual confrontation of the water flow and the grout against each other to form a large number of water cavities on the upper side of the fissure and the bypassing flow of the grout to the lower sensor, No. 3, the pressure is elevated. Overall, the further away from the grouting hole, the less pronounced the decrease in pressure values, and the amount of deposited grout gradually decreases.

**Fig. 17:**
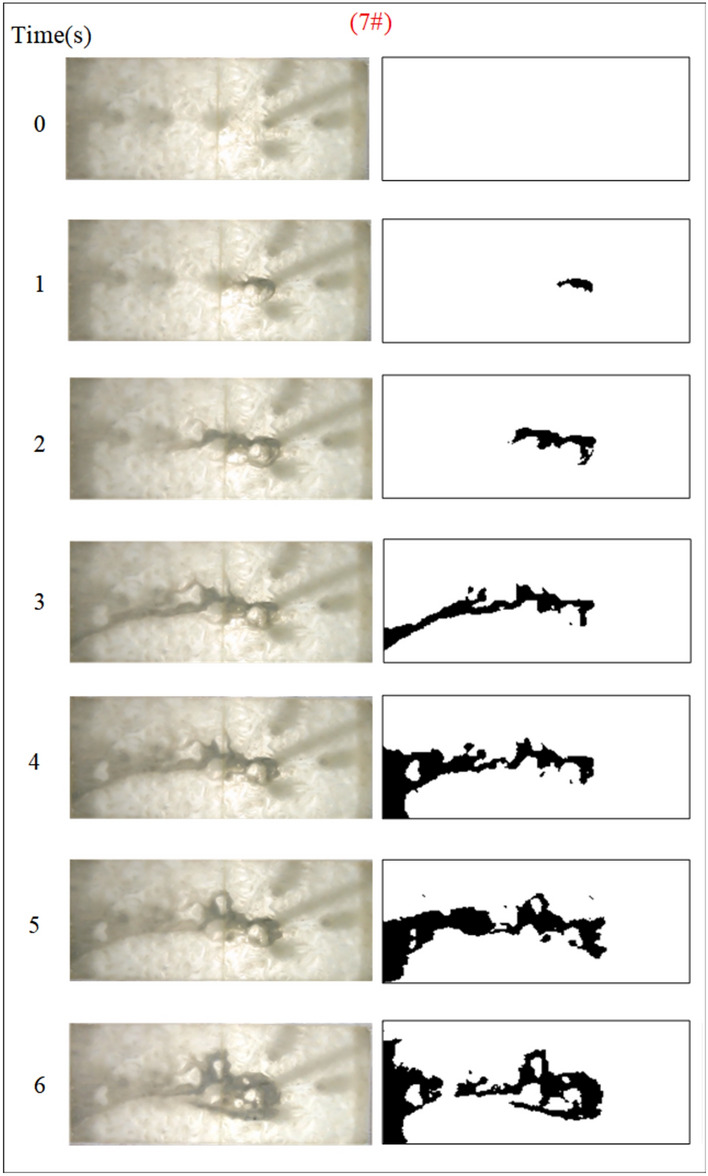
Grout diffusion patterns at Q_J_ = 0.06L/s.

**Fig. 18:**
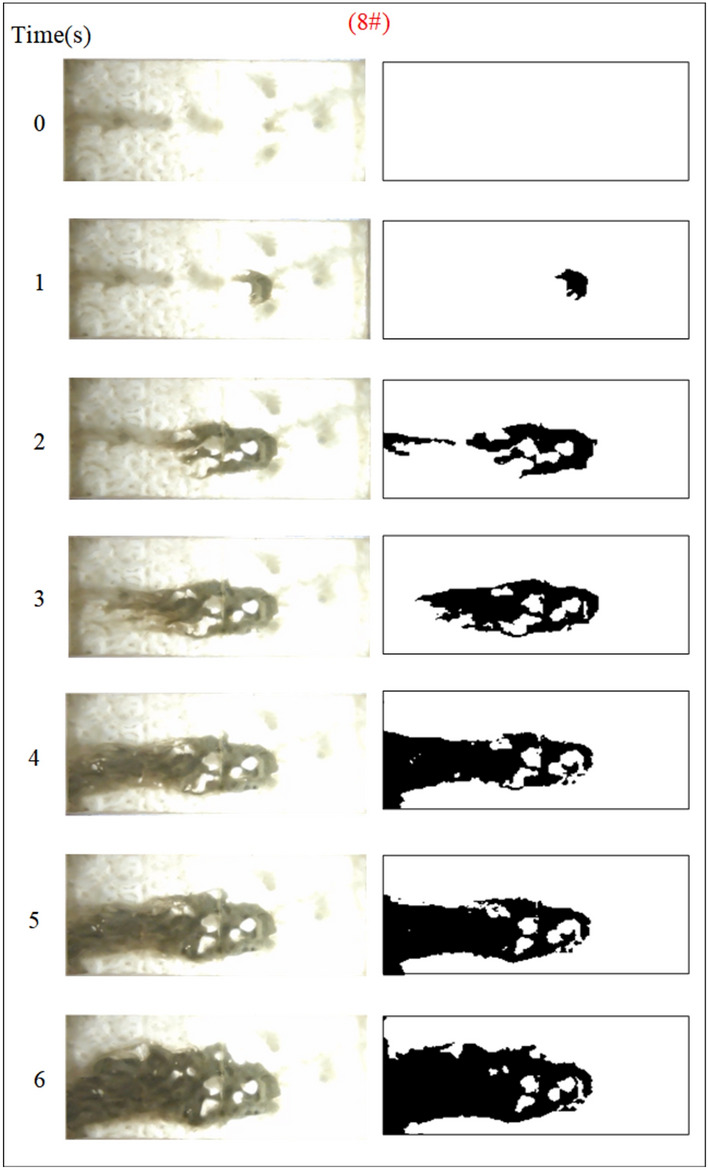
Grout diffusion patterns at Q_J_ = 0.08L/s.

**Fig. 19:**
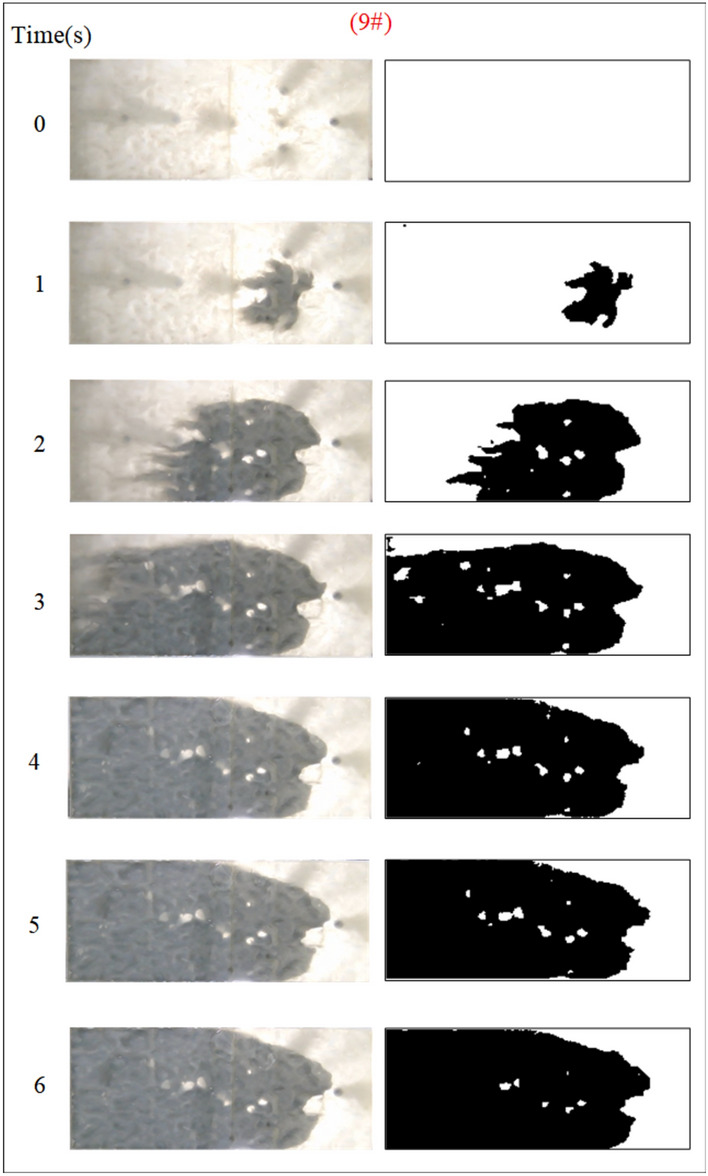
Grout diffusion patterns at Q_J_ = 0.10L/s.

**Fig. 20:**
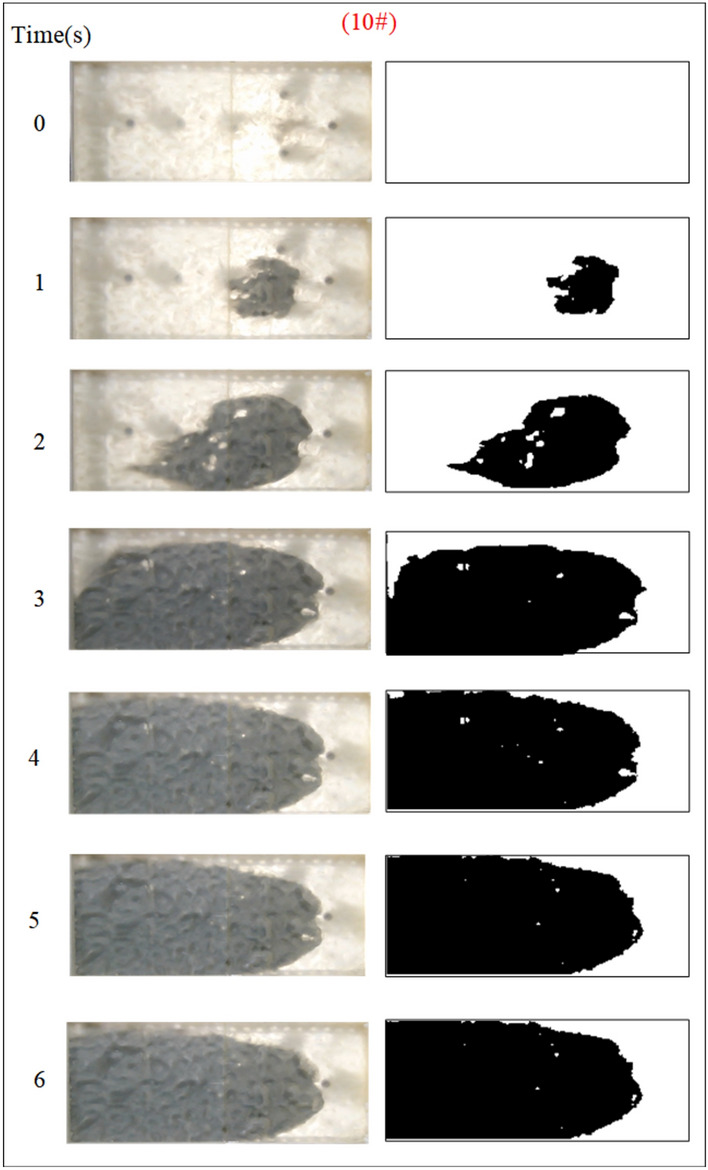
Grout diffusion patterns at Q_J_ = 0.12L/s.

Under the condition of guaranteeing that the rest of the parameters remain unchanged, the influence of the grouting flow rate on the spreading range and flow pattern of the grout is explored. The test numbers are 7#, 8#, 9#, and 10#, and the grout dispersion patterns are depicted in Figs. 17, 18, 19, 20. The grout is injected into the fracture; initially, the grout spreads radially in all directions; however, due to the roughness of the fracture, water cavity blisters are generated during the grouting process, and the grout has a roughly irregular circular shape. Grouting holes upstream in the grout and water flow under great confrontation generate a more uniform semicircular shape. The left and right sides of the grout away from the grouting hole, under the strong impact of the dynamic water impact, began to move downstream, and the speed of transport was greater than the center line near the grouting pressure under the action of the slurry diffusion speed. As the grout injection volume grows, the grout spreading upstream is constantly carried downstream by the flowing water, and due to the existence of the fracture boundary, the downstream grout continues to gather and fill the fracture.

According to the experimental results in Figs. 17, 18, 19, 20, it is found that the grouting diffusion pattern varies with the rise in grouting flow rate. In this research, after evaluating the link between the grouting flow rate and the dynamic water flow rate, it is divided into three types of diffusion patterns. (1) When the grouting flow rate is less than the dynamic water flow rate, it is difficult for the grout to be retained in the fracture, and a large amount of grout is washed away by the dynamic water only in the grouting hole and fracture at the end of the grout, and the diffusion between the two ends of the grout is in the form of an elongated streamline, and at this time, there is no effect of plugging the water at all; (2) When the grouting flow rate is equal to the dynamic water flow rate, the two sides of the formation of water passage are shown as a comet, resulting in the production of a large number of water bubbles in the water cavity; (3) When the grouting flow rate is more than the dynamic water flow rate, at this time, since the dynamic water flow rate is small, the grout in the fracture is in a flat state, which does not produce a wide range of confrontation with water. With an increase in time, the grout gradually fills the fracture. With the increase in the grouting flow rate of the water cavity, there is a gradual squeezed rupture, and the end of the fracture grout-water mixture outflow is getting smaller and smaller. In the downstream, the grout is filled by the grout, which is progressively transferred to the upstream.

**Fig. 21:**
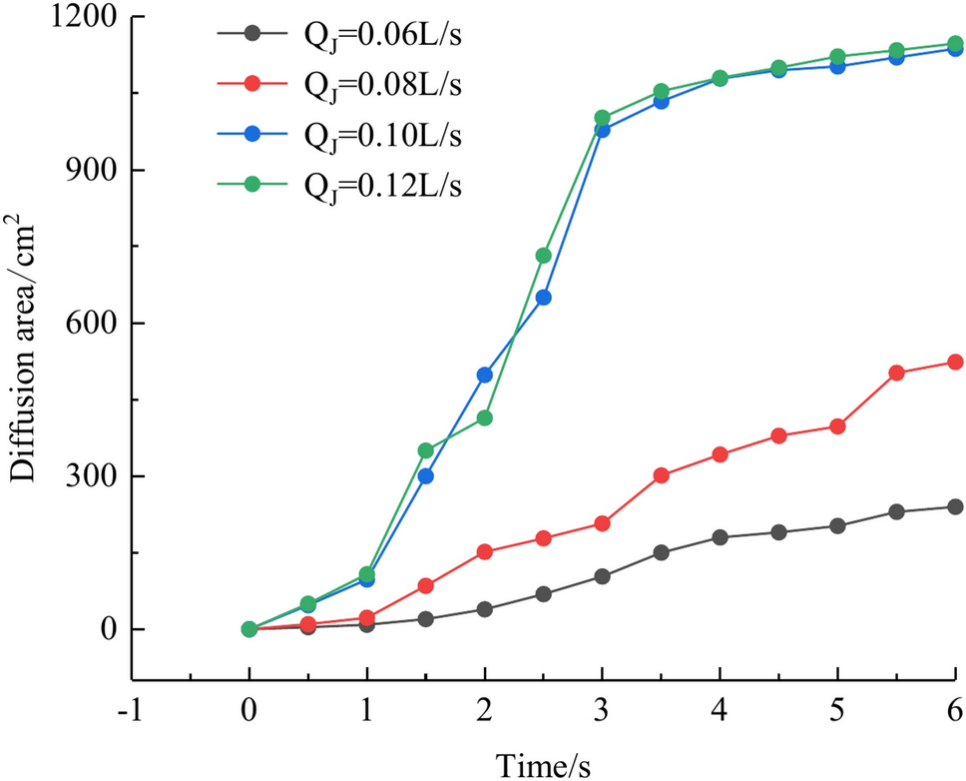
Spreading area of grout in the fracture under different grouting flow rates.

Based on the grayscale identification approach above, the grout diffusion area under varied grouting flow rates was computed as shown in Fig. 21. The maximal diffusion areas of the grouting flow rates QJ = 0.06 L/s, 0.08 L/s, 0.10 L/s, and 0.12 L/s are 240 cm^2^, 523.5 cm^2^, 1137 cm^2^, and 1147.5 cm^2^, respectively. The ratio of the maximum and minimum grouting diffusion area under different grouting flow rates is 4.78, and the enhancement of the grouting flow rate can greatly increase the spreading range of the grout to achieve the effect of water plugging. The test results show that when the grouting flow rate is less than the dynamic water flow rate, the grout diffusion area is not more than half of the total area of the fracture, and there are a large number of water cavities and blisters within the coverage of the grout, so it is not possible to effectively plug the water. Grouting flow rates greater than or equal to the dynamic water flow rate, the grout diffusion area being more than half of the total area of the fracture, and grouting pre-existing a large number of water cavities with the extension of time gradually disappearing can do effectively water plugging.

**Fig. 22:**
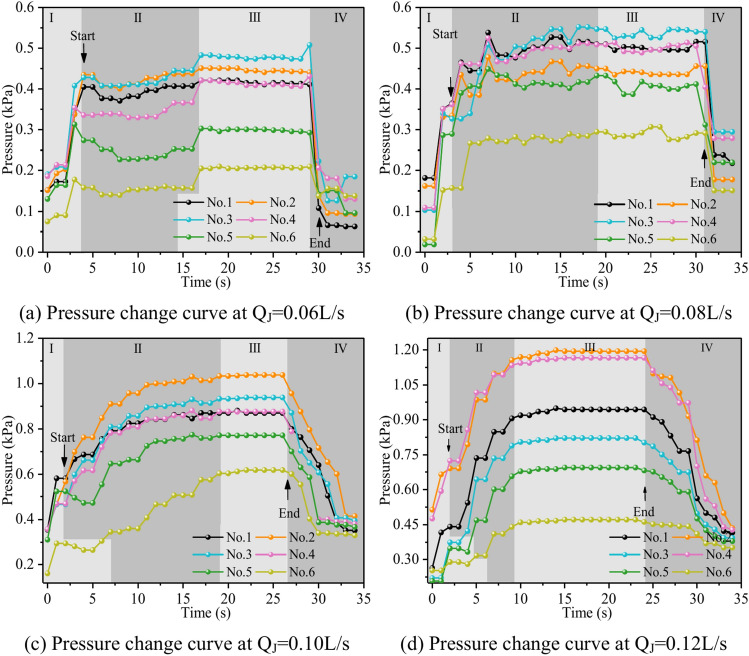
Pressure data of each monitoring point under different grouting flow rates.

Figure 22 shows the fluctuation of fluid pressure within the fractures throughout the grouting of Cases 7# to 10#. The peak pressures in the fractures for the injection flow rates Qw = 0.06L/s, 0.08L/s, 0.10L/s, and 0.12L/s are 0.508 kPa, 0.546 kPa, 1.038 kPa, and 1.194 kPa, respectively. From Fig. 22, it can be shown that, in general, the greater the grouting flow rate, the smaller the fluctuation range of the pressure curve in the fissure, and the pressure value increases. The reason is that, with the increase in grouting flow rate, the grout and water in the fracture intensified, but the effective deposition of grout to close the fissure grew rapidly, so there was a “smooth” rise in the pressure value phenomenon. When the grouting flow rate is greater than or equal to the dynamic water flow rate, the diffusion pattern is virtually the same as that of Case 1# (smooth fracture), with the difference that a small number of water cavities occur inside Case 9# and 10# (rough fracture). When the grouting flow rate is less than the dynamic water flow rate, the upstream grout and water impede each other, while the downstream water flow is accelerated by the grout to take away, generating an elongated streamline or comet shape, as seen in Figs. 17 and 18. When the grouting flow rate is greater than or equal to the dynamic water flow rate, at the upstream interface, the movement of water and grout cancels each other out and enters a stationary state. The grout first gels at the front face, where the flow velocity is lowest, and a retention nucleus (stagnation area) is created. The grout circulates in the vicinity of the stagnation area and then continues to cement. As the area of cementation expands and eventually reaches the boundary of the fracture, water flow is blocked, as shown in Figs. 19 and 20. Taking the grouting flow rate of 0.12L/s as an example for analysis, the peak pressures of Nos. 1 ~ 6 sensors are 0.944 kPa, 1.194 kPa, 0.822 kPa, 1.166 kPa, 0.695 kPa, and 0.471 kPa, respectively. Due to the conflict between the grout and the water, the grout flows around sensor No. 4, and the particles continue to deposit, resulting in a large pressure rise.

**Fig. 23:**
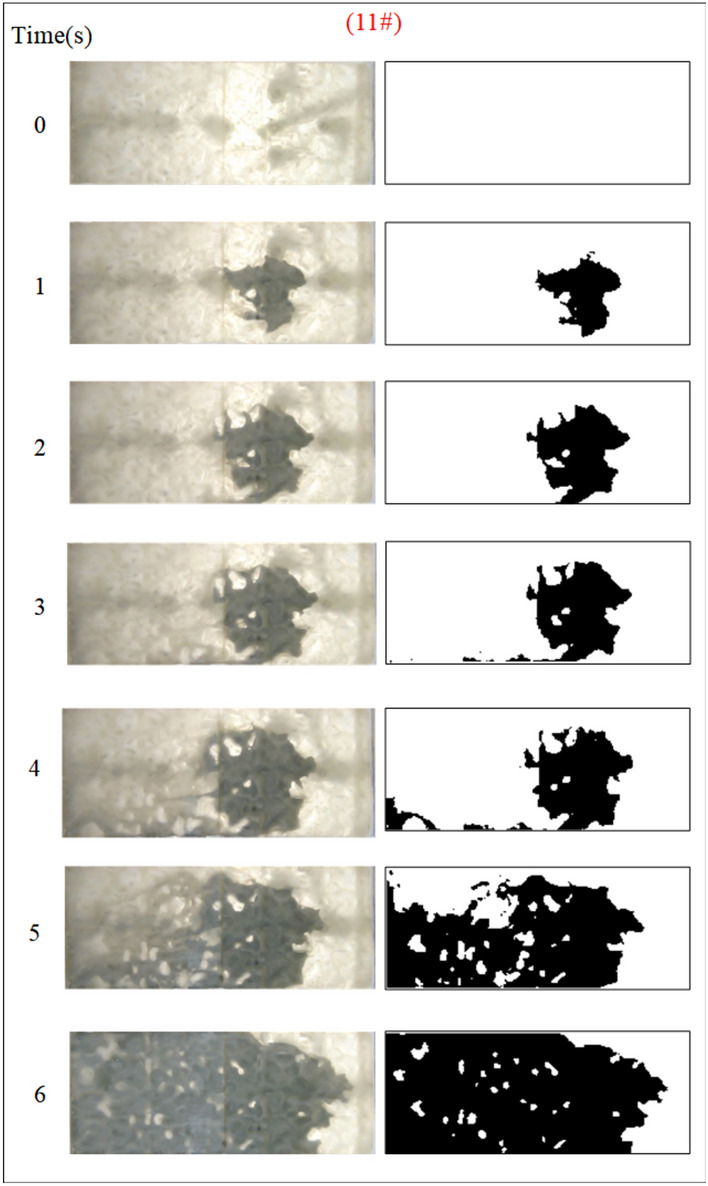
Grout diffusion patterns at W/C = 0.6

**Fig. 24:**
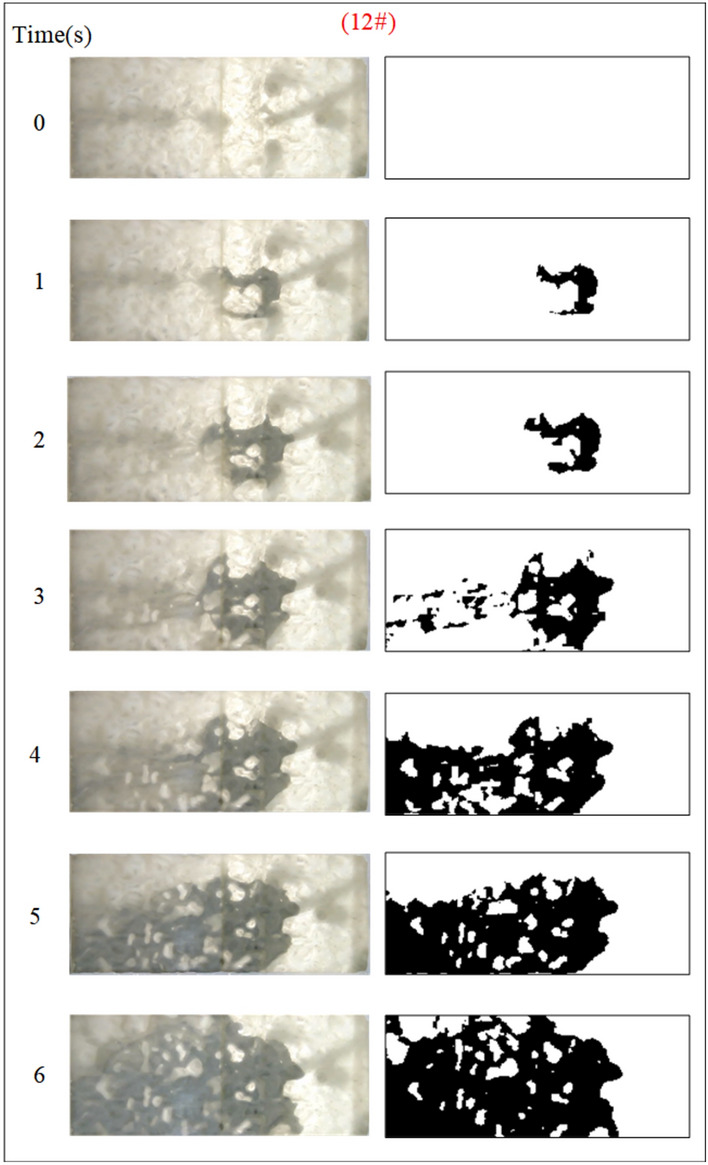
Grout diffusion patterns at W/C = 0.8

**Fig. 25:**
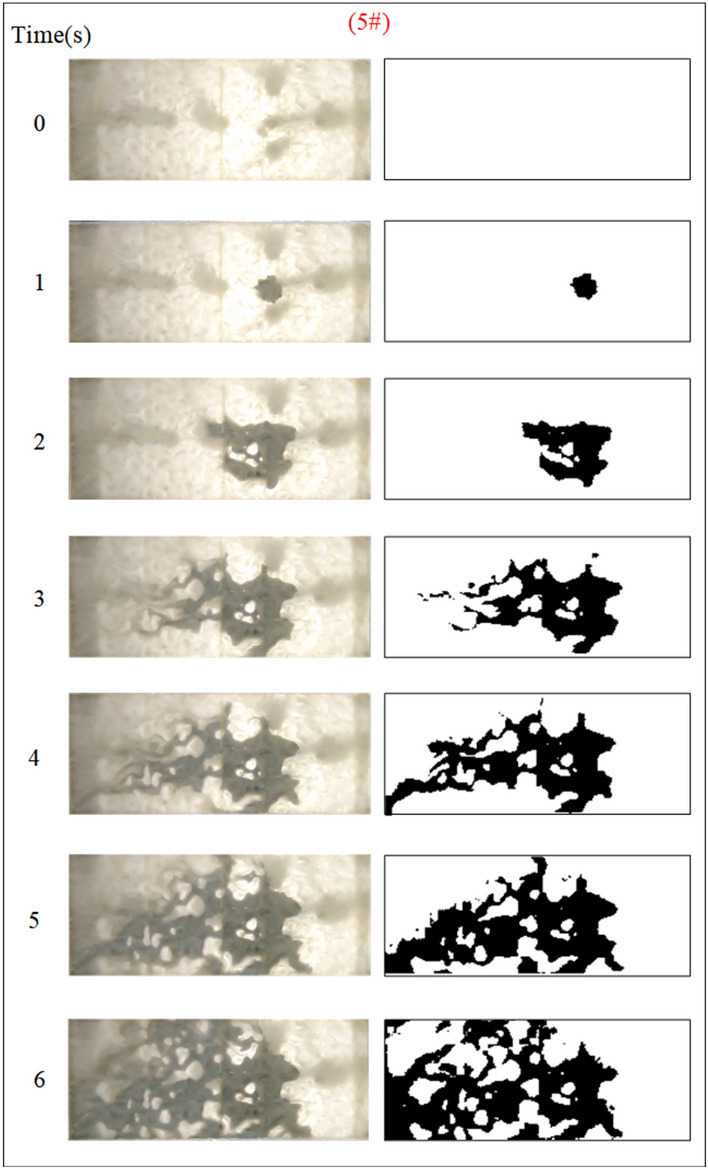
Grout diffusion patterns at W/C = 1.0

**Fig. 26:**
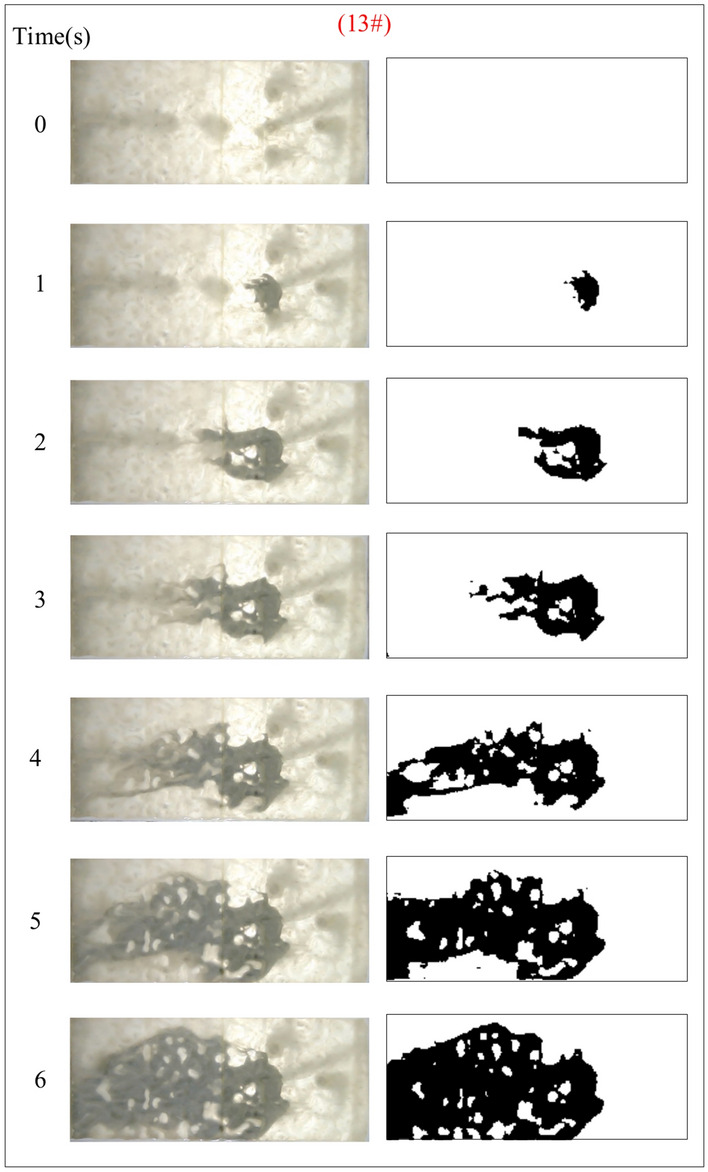
Grout diffusion patterns at W/C = 1.2

Under the condition of guaranteeing that the rest of the parameters remain unchanged, the influence of the grout water-cement ratio on the spreading range and flow pattern of the grout is explored. The test numbers are 11#, 12#, 5#, and 13#, and the slurry diffusion patterns are shown in Figs. 23, 24, 25, 26. After the grout is injected into the fracture, it spreads radially in all directions; however, due to the existence of fracture roughness, water voids are produced during the grouting process, and the grout spreading pattern is not uniform. As the grout injection time continues to rise, the grout spreading upstream continues to be carried downstream by the running water. The existence of the fracture boundary leads the downstream grout to constantly gather and fill the fracture, demonstrating a higher filling efficiency with a decreased water-cement ratio.

The grouting diffusion pattern changes as the water-cement ratio increases. According to the experimental phenomena in Figs. 23, 24, 25, 26, after analyzing the grouting diffusion patterns under different water-cement ratios, they may be divided into three categories of diffusion patterns. (1) When the water-cement ratio is 0.6, it is difficult for the grout to be cleansed by the running water, and its diffusion pattern is given as a cross-section type. At this moment, the effect of water plugging is good, and the flow rate of the grout-water combination at the exit of the fracture is very tiny; (2) When the water-cement ratio is between 0.8 and 1.0, on the two sides of the development of the water channel, the grout is displayed as a comet, resulting in the formation of water cavities; (3) When the water-cement ratio is 1.2, the grout and water form a wide spectrum of conflict, resulting in a high number of water cavities and blisters. The left and right sides of the slurry diffusion away from the grouting holes, subject to the strong impact of the water impact, began to travel downstream, forming a thin line, and eventually, the diffusion of morphology for the comet type and the effect of plugging were poor.

**Fig. 27:**
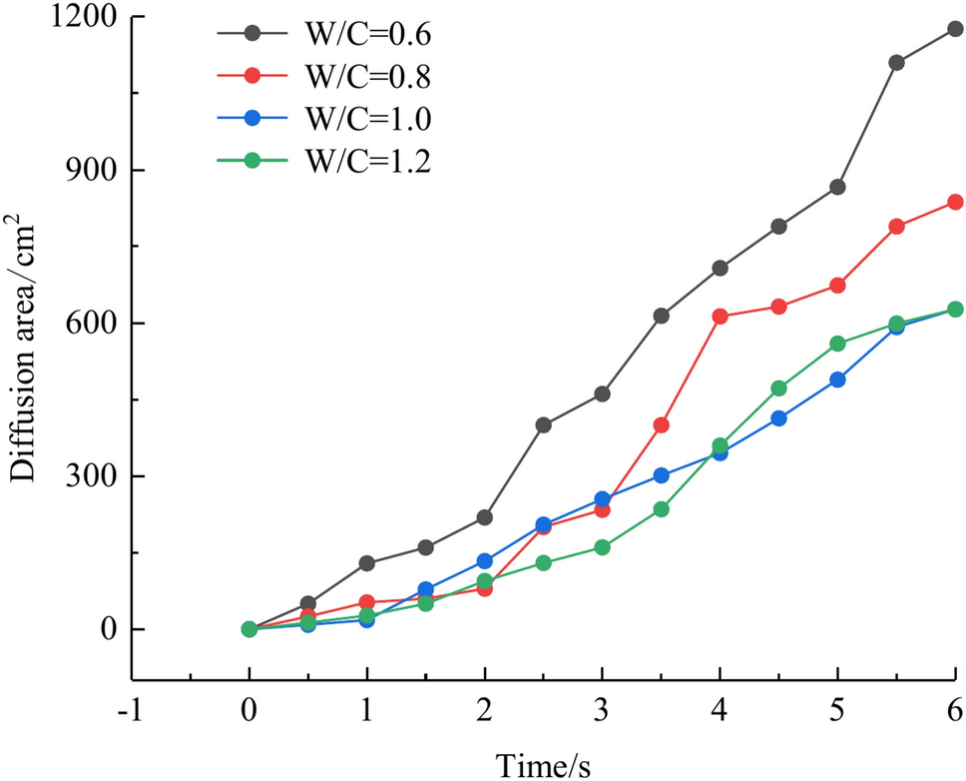
Area of grout diffusion in the fracture with different water–cement ratios.

We calculated the diffusion area of grout for various water-cement ratios using the previously indicated gray identification approach. This information is depicted in Fig. 27. The maximum regions of slurry diffusion were determined to be 1176 cm2, 837 cm2, 672 cm2, and 627 cm2 at grouting flow rates of W/C = 0.6, 0.8, 1.0, and 1.2 correspondingly. The ratio of the maximum area for slurry diffusion, which occurs at the minimum and maximum water-cement ratios, is determined to be 1.75. A decrease in the water-cement ratio results in a wider range of slurry diffusion, which is necessary for accomplishing successful water plugging. The test findings demonstrate that reducing the water-cement ratio leads to a wider range of grouting, but it also causes a significant increase in the pressure needed to accomplish the same grouting flow rate. In addition, a lower water-cement ratio results in a decrease in the production of cavity bubbles during the grouting process and greatly improves the effectiveness of water plugging.The grayscale recognition calculation results, shown in Figs. 24 and 25, indicate that there is minimal variation in the final slurry diffusion area between water-cement ratios of 0.8 and 1.0. Additionally, the early-stage grouting efficiency at a water-cement ratio of 1.0 exceeds that at a ratio of 0.8.

**Fig. 28:**
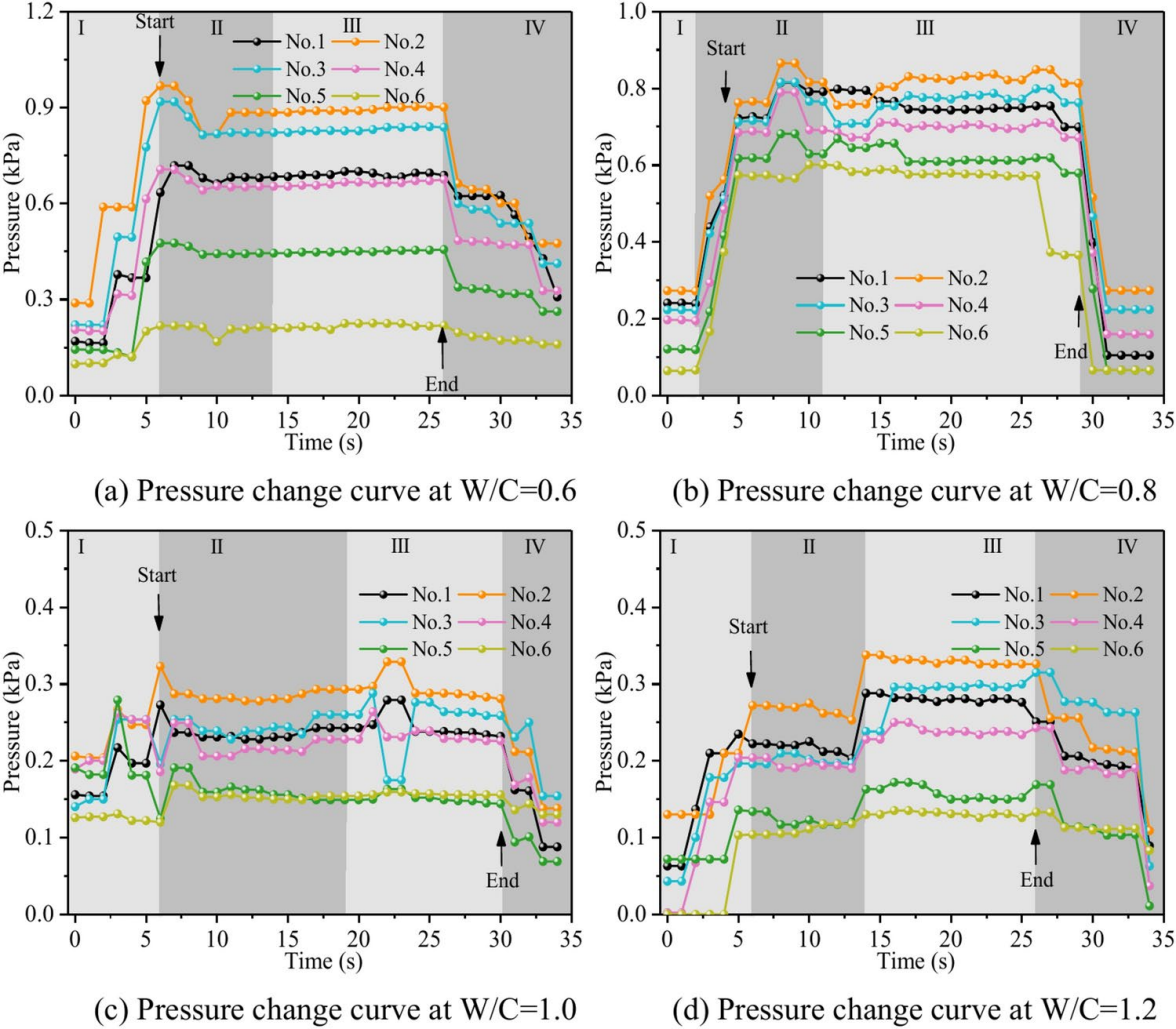
Pressure data at each monitoring point under different water–cement ratios.

Figure 28 depicts the fluctuation of fluid pressure within the fractures during the grouting of Cases 5# and 11 ~ 13#. When the water-cement ratios W/C are 0.6, 0.8, 1.0, and 1.2, the peak pressures in the fracture are 0.968 kPa, 0.849 kPa, 0.329 kPa, and 0.338 kPa, respectively. From Fig. 28, it can be seen that, in general, the smaller the water-cement ratio, the narrower the range of fluctuation of the pressure curve within the fracture, but the pressure value is higher. The explanation is that as the water-cement ratio lowers, the total mass of the grout particles per unit of time increases, leading to a quick increase in their effective deposition and a rapid increase in the pressure values. The analysis was carried out with an example of a water-cement ratio of 0.6, and the peak pressures of Nos. 1 to 6 sensors are 0.719 kPa, 0.968 kPa, 0.918 kPa, 0.706 kPa, 0.476 kPa, and 0.218 kPa, respectively. Due to the huge quantity of grout injection, it is difficult for the water to disperse the grout particles on a broad scale, and the grout is uniformly carried and diffused within the fracture. As a result, the No. 2 sensor closest to the position of the grouting hole has the biggest pressure value and the largest volume of grout deposited. While the other sites grow in distance from the grouting hole, the pressure value falls progressively, and the volume of grout poured similarly reduces sequentially.

## Water plugging mechanism

### Grouting-water adversarial modes

**Fig. 29:**
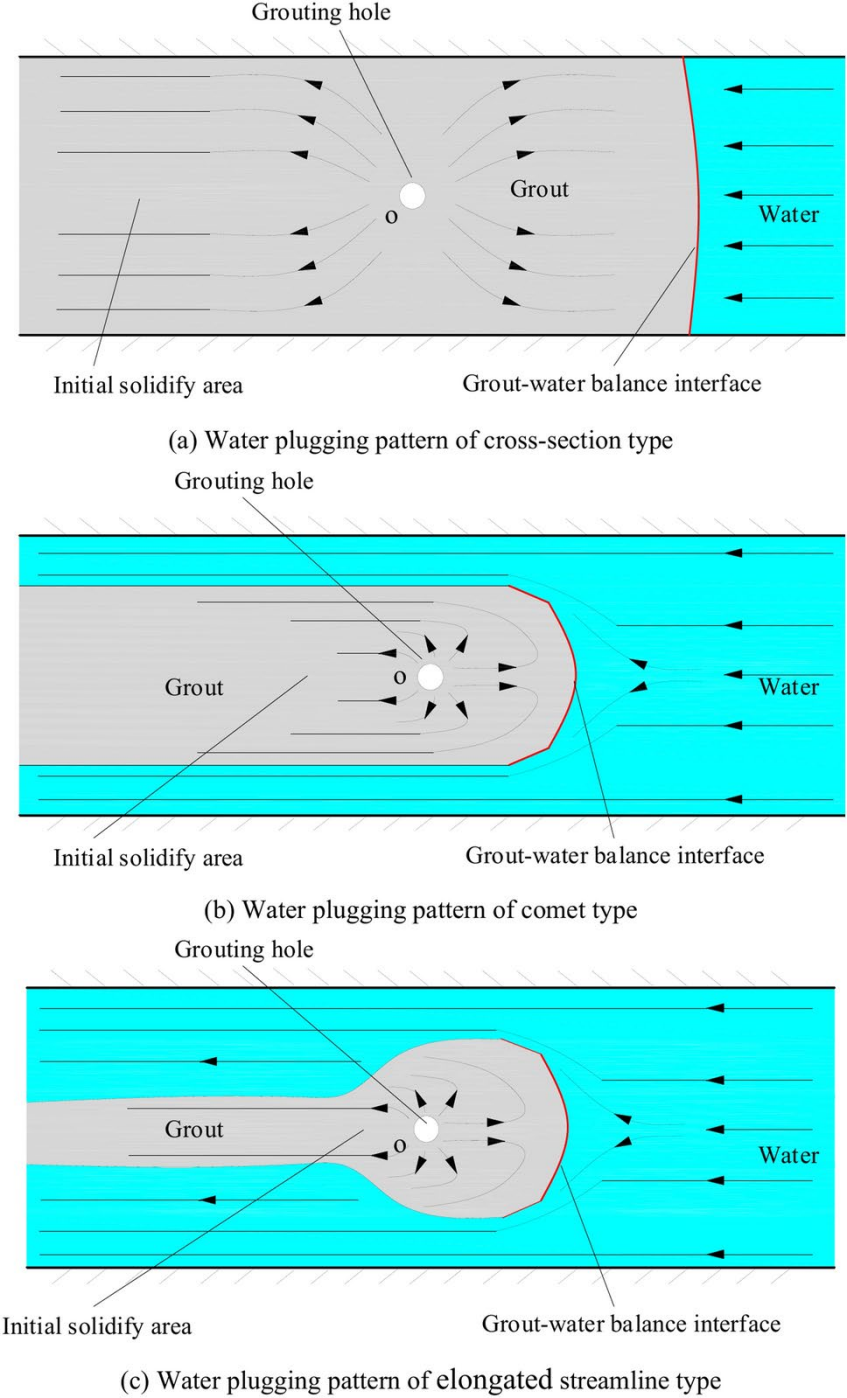
Classification of plugging patterns by Cases 1# ~ 13#

Factors such as the fractal dimension of the fractures, dynamic water flow rates, grouting flow rates, and water-cement ratios can all affect the diffusion and plugging process of the grout in the fracture in different forms and to different degrees. As shown in Fig. 29, the images of the final spreading pattern of the grout for each condition (Cases 1# ~ 13#) can be classified into the following three categories: (1) cross-section type; (2) comet type; and (3) elongated streamline type.

According to Fig. 29a, the rate at which grout flows is higher than the rate at which water flows. This causes the grout to enter the fracture, spread quickly, and fill the whole cross-section of the fracture, thereby blocking the flow of water. The interaction between grout and water will create a nearly vertical boundary. This disrupted boundary at the grout will be further diluted by the movement of water. Consequently, the accumulation of grout is less than that of the area downstream, which is not exposed to the movement of water. As depicted in Fig. 29b, when the rate at which grouting is injected is equal to or lower than the rate at which water flows dynamically, the grout and water counteract each other upstream, while the grouting pressure accelerates the downstream water, resulting in the formation of a comet-shaped structure. Under these conditions, the grout first gels at the front face, where the flow rate is minimized and a retention region is produced. The grout flows around the vicinity of the retention region and then continues to adhere. As the gelled area spreads and eventually reaches the perpendicular boundary of the fracture, the flow is stopped. If the grout does not spread to the boundary, the running water will continue to flow along both sides of the water passage. As demonstrated in Fig. 29c, a third elongated, streamlined structure is created under the impact of varied fractal dimensions and water-cement ratios. The first instant is identical to the second type of water-plugging structure; at the upstream fractal interface of water and grout, movement cancels each other out to reach a stagnant state. Grout flows around the stagnation nucleus, and due to the fractal dimension expanding or grout water-cement ratios increasing, it is difficult to continue to stay. Meanwhile, a huge number of grout particles are washed away by running water, and finally, the creation of water cannot properly plug an elongated streamlined structure.

### Grouting deposition mechanism with flowing water

Based on the experimental results, the dynamic diffusion process of grout in finite boundary fractures may be separated into two stages. In the first diffusion stage, after injection from the grouting hole, the grout distributes around the fracture but does not reach the fracture distribution border stage. At this stage, the grout diffusion speed upstream is small, while the downstream diffusion speed is rapid. At the interface between grout and water, the grout was diluted and cleaned by water. In the second diffusion stage, when the diffusion diameter of the grout is larger than the width of the fracture, the grout begins to cover the entire fault and diffuse upstream and downstream of the fracture due to the restrictions of the fracture boundary. Because the gelation time of the grout is long and affected by dynamic water pressure, the diffusion speed of the grout in the upstream direction is sluggish, and the diffusion speed in the downstream direction is quick. In practical grouting engineering, the distribution range of fracture is frequently extensive; thus, the law of grout-water flow without lateral border circumferential diffusion is ubiquitous.

**Fig. 30:**
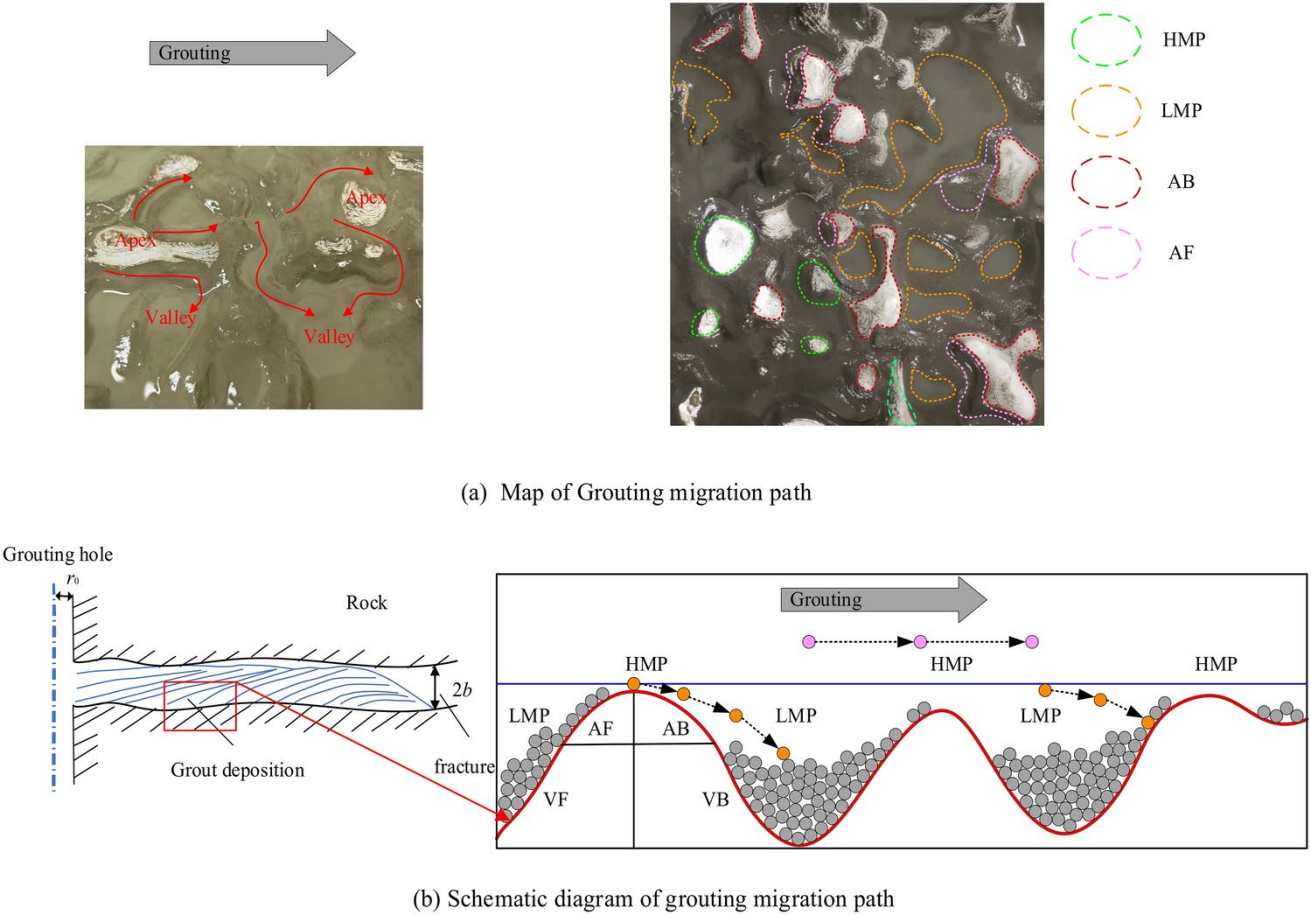
Mechanism of grout transport and deposition within a rough fracture.

Grouting in fine fractures: when the grout from the hole into the formation fissures with the continuous grouting cross-sectional area getting bigger and bigger, the grouting flow rate is inversely proportional to the length from the center of the grouting hole. The kinetic energy of the cement particles decreases due to the decrease in flow velocity. On the one hand, the sedimentation rate under gravity increases, and on the other hand, the cement particles are adsorbed by the rock wall they come into contact with, and the cement particles are attracted to each other to form a large group of particles, which facilitates the water separation and sedimentation process of the grout. Figure 30 depicts the effect of grout deposition during the grouting procedure. For a routable fracture, starting from the site of deposition, the cement particles will be detached from the grouting flow one after another, forming an ever-thickening “ridge” in the fracture, which will make the aperture of the fracture smaller and smaller until it is eventually filled. The dynamic water grout plugging in rough fractures is split into three stages: (1) Due to the geometry of the rough fracture surface, the diffusion path of the grout is meandering and initially follows the advantage path, i.e., bypassing the apex position to the valley location in Fig. 30a. At the same time, the cement particles are deposited at the bottom of the crack valley by gravity to form a sedimentary layer. After the cement-based slurry begins to gel, water (including water precipitated by the grout and flowing water) forms a hierarchy with the grout deposit layer. However, the fracture space is still large enough to allow water to flow through it, and dynamic water can still flow over the sedimentary layer, which does not make an effective block. As the grouting process goes on, the thickness of the sedimentary layer continuously develops, and the water flow channels decrease. At the same time, the number of prominent channels of grout diffusion rose, and the width increased. At this time, part of the sedimentary layer established a plugging zone, which played a role in inhibiting the dynamic water. (2) There is a water-separating impact on the grouting diffusion, and the dynamic water capture caused by the interaction of grout and water leads to the fast precipitation of free water in the grouting. It is worth highlighting that the water separation of the grout and the scouring action of the running water led to a steady reduction in the concentration of the sediment layer from bottom to top, and the distribution of the concentration of the sediment layer was not uniform. (3) As the grout continues to be deposited, the sedimentary layer covers most of the fracture surface, and some of the grout diffusion channels shift from curved to straight. At this time, the influence of the rough cleft surface on the grout diffusion is minimized, and the flow space of the fluid in the rough cleft is approximated to the smooth cleft. This means that the flow status of the grout and water is substantially altered.

To better understand the transport and deposition processes of the grout in the rough fracture, this paper divides the rough interface region into four regions, namely, apex front (AF), apex back (AB), valley front (VF), and valley back (VB), and analyzes its deposition characteristics, as shown in Fig. 30. As can be seen in Fig. 30a, the amount of grout deposits deposited in the apex zone is less than in the valley zone. This can be explained by the flow characteristics of the rough fracture surface, as revealed by Marchis et al.^33^, where the average velocity of the outer layer is uniform in rough fracture. On the contrary, close to the rough wall site, the velocity distribution is modified by the geometry of the rough fracture surface. Due to the difference in roughness height^34^, low-momentum (LMP) and high-momentum (HMP) routes alternate in the roughness fracture, as shown in Fig. 30b. This intermittent activity is related to the apex and valley; particularly, high-speed regions are dispersed in the apex while low-speed sections are scattered in the valley. This intermittent activity is related to the apex and valley; particularly, high-speed regions are dispersed in the apex while low-speed sections are scattered in the valley. Under these flow conditions, the cement particles in the apical region are easily washed away by the quick grout-water mixing flow^35^. However, as the cement particles enter the valley zone, which can readily be peeled off by LMP, gravity, and the contact of the cement particles with the fracture surface, more grout is deposited over time in the trough region. In addition, in the crest region, more grout adheres to the apex front (AF) than to the valley back (AB). This is because, under the combined influence of grouting pressure and flowing water, the slurry will diffuse across the flow in front of the peak to find an advantageous path, resulting in cement particles that are not easily deposited against the direction of grout diffusion.

Assuming that the rock fracture is a cavity without filler, in the process of grout particles being deposited to form a “ridge” impacted by the particles and water, the rock fracture porosity change can be stated as follows

1 $$n = 1 - \theta \delta$$

where *θ* is the water-induced expansion coefficient; and *δ* is the volume of trapped particles in the rock fracture per unit volume.

Based on the percolation theory, the one-dimensional process of retention of cement particles in the voids is shown in the following equation, Eq. (2) is the clarification filtration equation and Eq. (3) is the continuity equation.

2 $$\frac{\partial C}{{\partial z}} = - \lambda C$$

3 $$\frac{\partial C}{{\partial z}} + \frac{\partial q}{{v_{s} \partial t}} = 0$$

where *C* is the volumetric concentration of cement grout, *λ* is the filtration coefficient, *q* is the amount of cement particles stored per unit volume, and *v*_*s*_ is the filtration rate.

Substituting Eq. (2) into Eq. (3) and integrating over it, the porosity changes as follows

4 $$n = n_{0} - \int_{0}^{t} {\lambda v_{s} C} {\text{d}}t$$

where *n*_0_ is the initial porosity, which is 1 when there is no filler in the fracture.

According to the law of conservation of mass, the material migration transport equation for grout particles is as follows

5 $$\frac{\partial q}{{\partial t}} + \frac{{\partial \left( {nC} \right)}}{\partial t} = \frac{\partial }{\partial z}\left( {D_{h} \frac{{\partial \left( {nC} \right)}}{\partial z}} \right) - \frac{{\partial \left( {v_{s} C} \right)}}{\partial z}$$

where *n* is the porosity and *D*_*h*_ is the dispersion coefficient.

Substituting Eqs. (2) and (3) into Eq. (5) yields

6 $$v_{s} \lambda C + \frac{{\partial \left( {nC} \right)}}{\partial t} = \frac{\partial }{\partial z}\left( {D_{h} \frac{{\partial \left( {nC} \right)}}{\partial z}} \right) - \frac{{\partial \left( {v_{s} C} \right)}}{\partial z}$$

## Discussions

In the actual grouting project, due to its hidden nature, it is not possible to directly and effectively observe the grouting process in real-time, and only indirect indicators such as grouting pressure, time, volume, and changes in water influx can be used as grouting stopping criteria. When using the grouting method for surge water sealing and reinforcing the rock body, the two most important indicators are grouting water plugging and the reinforcing effect. In the dynamic water grouting test, the dynamic diffusion pattern of the grout may be monitored in real time during the operation, so it is proposed to use the grout diffusion area as an assessment index of the water plugging impact. The larger the grout diffusion area, the larger the space occupied by the grout in the fracture, the smaller the space accessible for the flow of groundwater, and the better the water-blocking effect. Define the grout plugging area ratio (*S*_r_) as the ratio of the grout diffusion area to the fracture area when the grout diffusion area reaches the maximum area, which can be represented as

7 $$S_{r} = \frac{{S_{g} }}{{S_{f} }}$$

where *S*_g_ is the grout diffusion area and *S*_f_ is the fissure area.

The water plugging area ratio is determined according to Eq. (7). According to the test phenomenon and calculation results of each working situation, the effect of grouting water plugging may be categorized into three grades: Sr ≥ 0.7 (good), 0.7 > Sr ≥ 0.5 (middle), and Sr < 0.5 (bad). From the calculation results, it can be shown that the maximum plugging area ratio of the slurry under each working condition is 0.784 and the minimum plugging area ratio is 0.160.

**Fig. 31:**
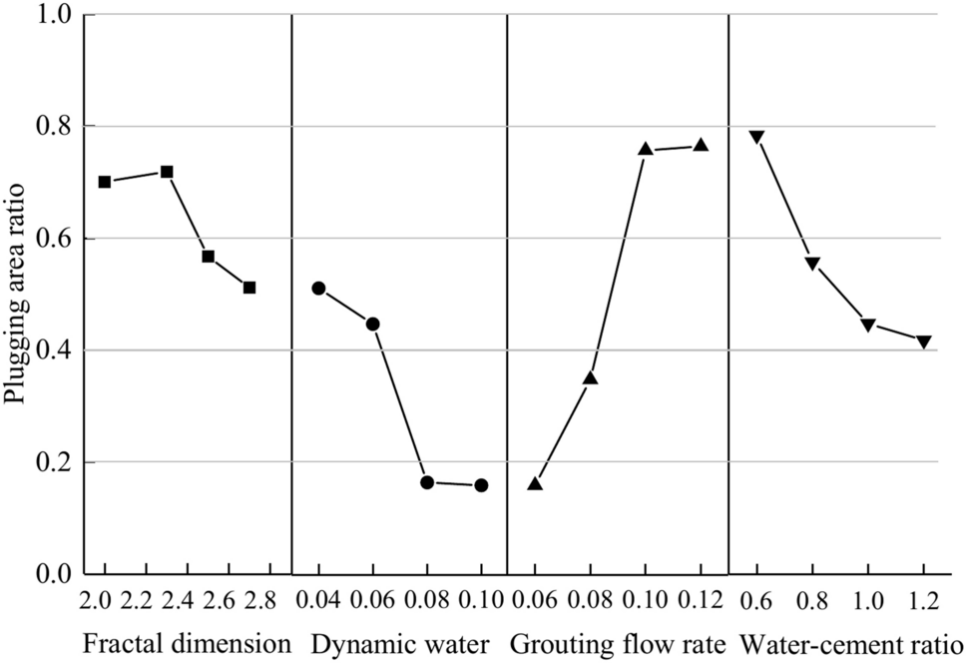
Distribution of water plugging rate under each working condition.

The distribution of fracture plugging area ratios under the action of different working circumstances was plotted, as illustrated in Fig. 31. Overall, the plugging area ratio is inversely proportional to the fractal dimension, dynamic water flow rate, and water-cement ratio, and positively proportional to the grouting flow rate. Of the 13 working situations, the grouting impact was bad in 5 cases, accounting for 38.4% of the total, while the grouting effect was moderate or above in 8 cases, accounting for 61.6% of the total.

The larger the fractal dimension of the fracture, the grout injected into the fracture will flow beyond the internal apex position to the valley position, and the grout will flow towards the outlet under the force of flowing water. During the diffusion process, the grout particles are deposited by gravity at the bottom of the valley, and free water is separated in the upper layer, generating an over-water layer in the upper layer of the grout. And then grout is injected by the original dynamic water scouring action, and grout in the water layer cannot continue to be effectively deposited, which is the main reason for the low grouting efficiency. In the actual engineering encounter with a fractal dimension larger fracture, one should increase the amount of grouting first to fill the fracture within the valley position or increase the grouting pressure to reduce the criss-crossing internal fractures to reach as close as possible to the smooth flat state so that the grout can fill the fracture layers layer by layer to increase the sealing effect of the grouting purpose.

Starting water flow is one of the important elements impacting the efficiency of water plugging, and for water flow rate and grouting flow rate, there is a competing mechanism. A higher dynamic water flow rate will increase the spreading rate of the grout, making the thickness of the inflowing grout thinner and less likely to be deposited in the fracture. Excessive dynamic water flow may even wash away some of the grout’s blocking portions after the grout has hardened, generating broad over-water tunnels and making the grout less effective at stopping the water flow. Reducing the size of the water flow rate is the most beneficial technique to increase the effectiveness of the blocking. Too high a dynamic water flow rate will prevent the grout from being kept on the fissure, and too low a flow rate will result in a shorter spreading of the grout and a shorter plugging length. In the real project, when the groundwater dynamic water flow is significant, it can be implemented using extra grouting holes, diversion, or first injection into the aggregate to minimize its flow. When the dynamic water flow rate is small or even has no water flow influence, the fluidity of the grout can be improved by increasing the grouting pressure or selecting the grout with a lengthy gelling time to boost the plugging effect. Suitable grouting materials can also be selected as needed to improve the water-plugging effect.

The initial grouting flow rate is a significant component impacting the efficiency of water plugging. A larger grouting flow rate will accelerate the diffusion rate of the grout, and at the same time, the continuous injection of grout will cause the thickness of the deposits in the fracture to consistently thicken. When the grouting flow rate is small, it will slow down the diffusion rate of the grout, and the thickness of deposition in the fracture will be weakened under the constant flushing influence of the external dynamic water, which will cause the effect of grouting plugging to worsen. With other variables being the same and increasing grout flow rate, the difference in plugging area ratio between 0.10 L/s and 0.12 L/s is not significant. The implication of this for practical engineering is that the grouting flow rate cannot be increased without an upper limit and that there is a threshold value that is reached in practical grouting projects where further increases in the grouting flow rate will not be beneficial to the increase of the blockage area within the fracture.

The water-cement ratio is a controllable component in grouting projects; its value is self-evident. The smaller the water-cement ratio, the greater the grout concentration, and the more grout particles may be injected per unit of time. Conversely, fewer grout particles can be injected per unit of time. The foregoing behavior is reflected in the test, as the smaller the water-cement ratio, the bigger the grouting blocking area ratio. However, there is a problem: the smaller the water-cement ratio, the viscosity of the grout itself will continue to increase with time. For the actual project, it is difficult to provide a large enough grouting pressure in the fracture rock body pumped into the small water-cement ratio grout. Therefore, it is very vital to configure the grout water-cement ratio fairly to achieve optimal grouting efficiency for the real project.

When the grout flow rate is less than or equal to the water flow rate, the upstream grout and water flow retard each other, while the downstream water flow is accelerated and carried away by the grout, forming a teardrop-like structure. The movement of water and grout cancel each other out to a stagnant state at the upstream partition interface. The grout will first gel at the front face where the flow rate is minimal and form a retention nucleus (stagnation area). The grout flows around the retention nucleus and then continues to cement. As the area of cementation expands and eventually reaches the boundary of the fracture, the flow of water is blocked. If the grout does not travel to the boundary, the water will continue to flow along the water passage channels on both sides. The experimental phenomenon shows that the final diffusion pattern of grouting is closely related to the velocity of dynamic water and the velocity of grouting. In terms of engineering, it is more desirable to have cross-sectional grouting to cut off the flow channel.

## Conclusions

In a transparent dynamic water grouting test system constructed by 3D printing, the influences of fractal dimension, water flow velocity, grout flow velocity, and water-cement ratio on the grouting diffusion properties of fractured rock with flowing water were thoroughly examined. The primary conclusions from this study can be derived as follows:

1. Construct a 3D rough fracture surface based on fractional Brownian motion and utilize Rhinoceros software to actualize the generation of 3D data of the rough fissure surface and the establishment of the test model. A physical model of the rough fracture surface was generated using 3D printing technology to offer a model basis for the 3D rough fracture dynamic water grouting test. A visible dynamic water fracture grouting test set-up comprising a water source system, a grouting system, a data gathering system, and a fracture system was finally created.
2. A dynamic water fracture grouting test was carried out, which indicated that the morphology and mode of grout diffusion were affected by the fractal dimension of the fracture, the dynamic water flow rate, the grouting flow rate, and the water-cement ratio of the grout. The transport diffusion of grout within a finite boundary fracture (600 mm × 250 mm) can be separated into two stages, i.e., the stage of circumferential diffusion without a lateral boundary and the stage of diffusion along the boundary.
3. The diffusion pattern during grouting and plugging is divided into three types according to each operating condition: cross-sectional, comet, and elongated streamline. When the grout flow rate is greater than the dynamic water flow rate, at which point the grout enters the fracture, the grout will spread fast and cover the whole cross-section of the fracture, and the water flow is blocked. When the grouting flow rate is less than or equal to the dynamic water flow rate, the upstream grout and water hinder each other, while the downstream water is accelerated by the grout, generating a comet formation. When the grouting flow rate is less than or equal to the dynamic water flow rate, under the impact of varied fractal dimensions and water-cement ratios, a third elongated, streamlined structure is produced on the base of the comet structure. For the actual project, the dynamic water flow should be reduced and the success rate of water plugging should be increased.
4. The scouring influence of the moving water makes the grout diffusion speed rise in the direction of the flowing water, the diffusion area grows and stops the grout diffusion against the direction of the flowing water, and the diffusion area falls. Due to the rising amount of grout injected in the test, the overall trend of the fracture pressure field was upward, with high pressure near the grouting hole, and the farther away from the grouting hole, the greater the pressure loss and the smaller the pressure value. As the grouting portion continues to stretch, the pressure slowly decays from far to near.
5. In a coal seam saturated with water, it is necessary to select a high water-cement ratio, initial grouting rate, and grouting pressure while reducing the dynamic water pressure during the first grouting phase. This approach facilitates crack propagation and water expulsion from the coal seam. Once the grouting pressure stabilizes, the slurry can fully penetrate the fissures within the coal seam. By subsequently lowering the water-to-cement ratio, the viscosity of the slurry increases, accelerating the solidification reaction. This enables the slurry to rapidly gel on the crack surfaces, effectively sealing them and achieving the objectives of water blocking and reinforcing the water-rich coal seam.

## Data Availability

The [DATA TYPE] data used to support the findings of this study are available from the corresponding author upon request.
